# Versatile Hydrogel Dressing with Skin Adaptiveness and Mild Photothermal Antibacterial Activity for Methicillin‐Resistant Staphylococcus Aureus‐Infected Dynamic Wound Healing

**DOI:** 10.1002/advs.202206585

**Published:** 2023-02-12

**Authors:** Peng Zhao, Yu Zhang, Xiaoai Chen, Chang Xu, Jingzhe Guo, Meigui Deng, Xiongwei Qu, Pingsheng Huang, Zujian Feng, Jimin Zhang

**Affiliations:** ^1^ Hebei Key Laboratory of Functional Polymers School of Chemical Engineering and Technology Hebei University of Technology 5340 Xiping Road, Beichen District Tianjin 300130 P. R. China; ^2^ Tianjin Key Laboratory of Biomaterial Research Institute of Biomedical Engineering Chinese Academy of Medical Sciences and Peking Union Medical College 236 Baidi Road, Nankai District Tianjin 300192 P. R. China

**Keywords:** hydrogel dressing, mild photothermal antibacterial, MRSA infected wound healing, skin‐adaptiveness

## Abstract

Bacterial infection often induces chronic repair of wound healing owing to aggravated inflammation. Hydrogel dressing exhibiting intrinsic antibacterial activity may substantially reduce the use of antibiotics for infected wound management. Hence, a versatile hydrogel dressing (rGB/QCS/PDA–PAM) exhibiting skin adaptiveness on dynamic wounds and  mild photothermal antibacterial activity is developed for safe and efficient infected wound treatment. Phenylboronic acid‐functionalized graphene (rGB) and oxadiazole‐decorated quaternary carboxymethyl chitosan (QCS) are incorporated into a polydopamine–polyacrylamide (PDA–PAM) network with multiple covalent and noncovalent bonds, which conferred the hydrogel with flexible mechanical properties, strong tissue adhesion and excellent self‐healing ability on the dynamic wounds. Moreover, the glycocalyx‐mimicking phenylboronic acid on the surface of rGB enables the hydrogel to specifically capture bacteria. The enhanced membrane permeability of QCS enhanced bacterial vulnerability to photothermal therapy（PTT）, which is demonstrated by efficient mild PTT antibacteria against methicillin‐resistant *Staphylococcus aureus* in vitro and in vivo at temperatures of <49.6 °C. Consequently, the hydrogel demonstrate accelerated tissue regeneration on MRSA‐infected wound in vivo, with an intact epidermis, abundant collagen deposition and prominent angiogenesis. Therefore, rGB/QCS/PDA–PAM is a versatile hydrogel dressing exhibiting inherent antibacterial activity and has considerable potential in treating wounds infected with drug‐resistant bacteria.

## Graphical abstract

A versatile hydrogel conferred skin adaptiveness on dynamic wound healing, including flexible mechanical properties, strong tissue adhesion and excellent self‐healing ability. Moreover, the design of specific bacterial capture, membrane puncture and enhanced near‐infrared permeability, produced a safe and efficient mild PTT antibacteria against methicillin‐resistant *Staphylococcus aureus* and accelerated the tissue regeneration of MRSA‐infected wounds.

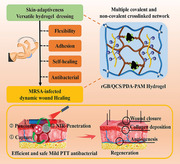

## 1 Introduction

Bacteria‐infected wounds derived from cutaneous trauma, burn and disease, remained a big challenge in global public health for high mortality rate and huge economic burden.^[^ ^1^ ^]^ During the process of wound healing, bacterial infection often lead to chronic inflammation, which significantly impeded the transformation to the subsequent proliferative and remodeling stage of wound healing, resulting in chronic wound healing or even failure to heal.^[^ ^2^ ^]^ However, the most commonly available treatments of antibiotics possessed numerous drawbacks, such as low bioavailability, side effects and especially drug resistance almost among all bacterial species.^[^ ^3^ ^]^ Among these, the most universal Methicillin‐Resistant *Staphylococcus aureus* (MRSA) infection,^[^ ^4^ ^]^ formed dense biofilm via special affinity between carbohydrate‐binding proteins (Lec A or Lec B) on bacterial surface and galactose on wound,^[^ ^5^ ^]^ resulting in damaging immune system of host defense and making increased difficulty for complete wound healing. Therefore, developing antibiotic‐free wound dressing for MRSA infected wound healing became more urgently needed.

Recently, various techniques with high antimicrobial efficiency, including photothermal therapy (PTT),^[^ ^6^ ^]^ photodynamic therapy (PDT)^[^ ^7^ ^]^ or chemo‐dynamic therapy (CDT),^[^ ^8^ ^]^ have received tremendous interest as alternative to antibiotics. Especially, the photothermal antibacterial therapy could convert light energy into heat and then thermal ablate bacteria via damaging intracellular oxidative stress, enzymatic activity, DNA lesion, meriting significant advantages of deep tissue penetration and feasible controlled photothermal temperature.^[^ ^9^ ^]^ However, efficient PTT antibacterial therapy commonly need high temperature (>60 °C), which was extensively higher than physiological temperature (37 °C), resulting in inevitable local thermal damages to normal cells and healthy tissues around wound.^[^ ^10^ ^]^ As per a previous report, low temperature (LT) less than 50 °C is a prospective range to prevent normal tissue from destruction by high temperature.^[^ ^11^ ^]^ While mild photothermal antibacterial (M‐PTT) effect was usually unsatisfied, for that bacterial apoptosis at these LT could be reversed via intracellular heat shock protein.^[^ ^12^ ^]^ Therefore, to address the contradiction of antibacterial efficiency and biosafety, it's a key issue to enhance the antibacterial efficiency of PTT at low temperature. Quaternary ammonium salt (QAS) is a traditional antibacterial agent, which possess antibacterial activity though destroying permeability of bacterial membrane.^[^ ^13^ ^]^ Thereinto, we hypothesized that integration of QAS to disrupt the bacterial membrane to make it more susceptible to PTT, this approach would benefit for improving the antibacterial efficiency of mild PTT.^[^ ^14^ ^]^ However, well coordinating the QAS and PTT in wound dressing to achieve high antibacterial effect and accelerate the infected wound healing, still remains uncertificated.

Hydrogel dressing had charming characterizations, including 3D structure, abundant water and tissue‐like solid–liquid properties, providing a moist and permeable environment for healing‐related cells proliferation and infiltration. Furthermore, hydrogel also solved the problem of lacking functionality of traditional wound dressings, such as inert bandage, felt pad and cotton gauze.^[^ ^15^ ^]^ Especially, the hydrogel dressing allowed for various feasible antibacterial functionalization to promote the wound healing.^[^ ^16^ ^]^ However, the current hydrogel dressing often suffers from various physicochemical drawbacks, including brittle mechanical properties, weak tissue adhesion and inferior self‐healing performance. These shortcomings greatly restricted the hydrogel application in dynamic wounds with high‐frequency movement, such as destructive wounds with large skin trauma and severe hemorrhage. As previously reported, multiple hydrogel network formed by chemical bond, and physical bond can efficiently balance the equilibrium of these inferiors.^[^ ^17^ ^]^ Strong chemical bond could boost mechanical strength, while physical bond could elevate self‐healing ability by rapidly reconstructing when the hydrogel had a breakdown. The co‐contribution of chemical and physical crosslinking further improved the toughness and adhesion, resulting in enhancing adhesion strength of the hydrogels.^[^ ^18^ ^]^ Therefore, the multiple‐network hydrogel dressing with non‐covenant and covalent crosslinking, provide the great promise to fulfill the requirements of dynamic wound management, including skin‐like mechanical strength, adhesiveness and tissue adaptiveness.

**Scheme 1:**
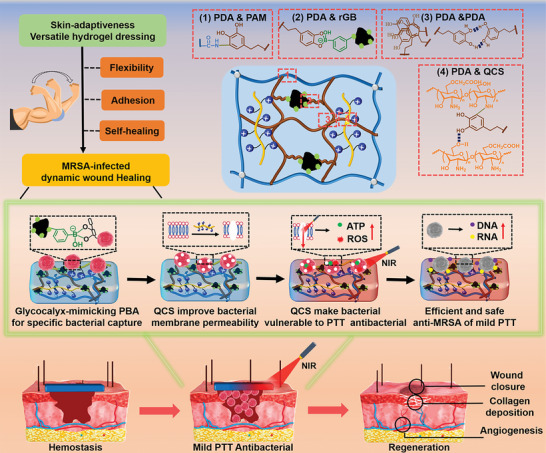
Schematic illustration of a versatile hydrogel dressing (rGB/QCS/PDA‐PAM hydrogel) with mild photothermal (M‐PTT) antibacterial performance for accelerating Methicillin‐Resistant *S. aureus*‐infected wound healing at low temperature (LT, <50 °C).

Herein, we rationally designed a multifunctional hydrogel dressing with tissue adaptiveness on dynamic wound and efficient antibacterial activity through mild photothermal effect for MRSA‐infected wound healing (**Scheme** **1**). The hydrogel (rGB/QCS/PDA‐PAM) was fabricated based on polydopamine (PDA) and polyacrylamide (PAM) crosslinking system, and further incorporated with photothermal 3‐aminophenylboronic acid modified reduced graphene oxide (rGB) sheets and QAS decorated carboxymethyl chitosan (QCS). We hypothesized that glycocalyx‐mimicking phenylboronic acid on rGB could first specific capture abundant bacteria on the surface of hydrogel. Then, QCS would puncture membrane of the trapped bacteria, and rGB would coordinate to achieve efficient antibacterial activity through mild photothermal effect at low temperature. Additionally, PDA acted as bridge to anchor flexible PAM chains, rGB, and QAS QCS for strengthen the crosslinking network. The multiple covalent and noncovalent bonds could endow the hydrogel dressing with flexible mechanical strength, strong adhesiveness, and good self‐healing performance to adapt the dynamic wounds with high frequency movement. When the hydrogel application as wound dressings in vivo, it was expected to inhibit the MRSA‐infection and accelerate the infected dynamic wound healing.

## 2 Results and Discussion

### 2.1 Synthesis and Characterization of rGB and QCS

**Figure 1:**
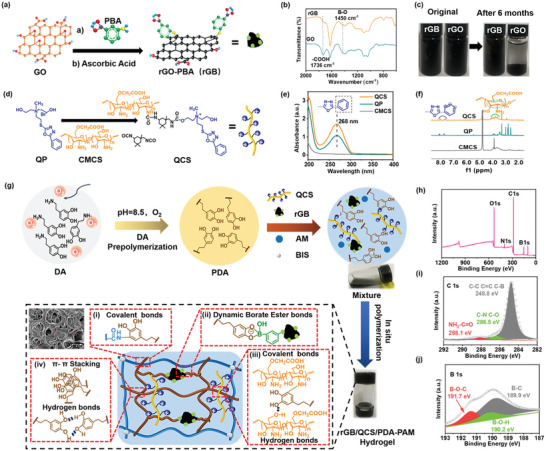
Synthesis and characterization of rGB, QCS, and rGB/QCS/PDA‐PAM hydrogel. a) Synthesis route of rGB nanosheet. b) FT‐IR spectra of GO and rGB. c) Dispersion stability of rGB and rGO in deionized water after 6 months. d) Synthesis route of QCS. e) UV absorption spectra and f) ^1^H NMR spectra of QCS, QP, CMCS. g) Preparation process and multiple covalent and non‐covalent cross‐linked network in rGB/QCS/PDA‐PAM hydrogel. XPS spectra of rGB/QCS/PDA‐PAM hydrogel with h) wide‐scan spectrum, i) C1s deconvolution, and j) B1s deconvolution.

The NIR‐triggered photothermal conversion efficiency of reduced graphene oxide (rGO) is higher than that of graphene oxide (GO), making 2D inorganic rGO sheets a favorite photothermal antibacterial candidate.^[^ ^19^ ^]^ Phenylboronic acid is perceived as a glycocalyx‐mimicking molecule that can specifically bind with diol‐containing saccharides on bacteria surfaces via boronate ester formation.^[^ ^20^ ^]^ Therefore, the rGO surface was decorated with glycocalyx‐mimicking phenylboronic acid to improve its ability to capture bacteria.^[^ ^21^ ^]^ The epoxy group on the surface of GO reacted with the primary amine of phenylboronic acid and was subsequently reduced by ascorbic acid to obtain phenylboronic acid‐decorated rGO (termed as rGB) (**Figure** **1**a). FT‐IR results showed that a decrease in —COOH stretching at 1736 cm^−1^ was attributed to GO reduction and the characteristic peaks presented at 1450 cm^−1^ belonged to the B—O stretching of PBA (Figure 1b).^[^ ^22^ ^]^ In UV–Vis absorption spectra (Figure S1a, Supporting Information), a characteristic peak at 296 nm attributed to PBA in rGB samples demonstrated successful preparation of rGB. For Raman spectroscopy (Figure S1b, Supporting Information), compared with the results for the initial GO material, the G‐featured peak of rGB shifted from 1582 to 1576 cm^−1^ while the chemical shift of the D‐featured peak remained unchanged. The calculated ratio of the intensities of the two peaks (*I*_D_/*I*_G_) increased from 0.939 for GO to 1.081 for rGB, depicting the reduction from GO to rGB.^[^ ^23^ ^]^ Notably, the obtained rGB suspended solution exhibited a dark gray color and had a well‐dispersed state that could be maintained for six months. Conversely, for the rGO sheets obtained by direct reduction without PBA modification, sedimentation appeared after six‐month storage (Figure 1c). PBA modification on the surface of ultrathin nanosheets avoided agglomeration induced by high surface energy, improving rGB stability.

QAS have attracted increasing attention in antibacterial dressings for contact‐damaging of bacterial plasma membranes. However, the antibacterial activities of traditional long chain alky‐QAS are contradicted by their poor biocompatibility as increasing alkyl‐chain length increases the cell toxicity induced by lipid‐soluble long alkyl‐chain insertion into cell membranes. 5‐Phenyl‐1,3,4‐oxadiazole–QAS (QP), a new type of QAS with phenyl substitutions instead of long alkyl chains, simultaneously guarantees high antibacterial activity and favorable biosafety.^[^ ^24^ ^]^ To improve the QP loading content (Figure 1d), QP was conjugated onto carboxymethyl chitosan (CMCS) with isophorone diisocyanate, the obtained polymer denoted as QCS. UV spectra tentatively defined successful QP grafting onto CMCS by a new peak at 268 nm in QCS, which was attributed to the absorption of benzene in QP (Figure 1e). FT‐IR spectra also confirmed the success of QCS synthesis, where, in comparison with the CMCS spectrum, new peaks were present at 1314 and 1612 cm^−1^ in the QCS spectrum, which were attributed to C—N vibration and benzene ring vibration in QP (Figure S2a, Supporting Information).^[^ ^25^ ^] 1^H NMR was employed to determine the chemical structure of QCS and QP substitution degree. The peaks at 7.95–7.56 ppm were attributed to phenyl protons of QP and those at 4.35–3.24 ppm were ascribed to the C2–C6 protons of CMCS glycan units.^[^ ^26^ ^]^ Based on the integration area, the QP substitution degree was ≈16.6% (Figure 1f). Notably, adjusting the QP/CMCS feeding ratio from 0.2 to 2.0 changed the QP substitution degree from 5.6% to 16.6%. The antibacterial efficiency of QCS depends on its positive potential, and the QP substitution degree of 16.6% with the highest potential (13.7 mV) was chosen for the subsequent studies (Figure S2b, Supporting Information).

### 2.2 Preparation and Structure Characterization of rGB/QCS/PDA‐PAM Hydrogel

The antibacterial rGB/QCS/PDA–PAM hydrogel dressing was prepared by simply incorporating the NIR‐triggered photothermal rGB and intrinsic antibacterial QCS into the PDA–PAM crosslinking system (Figure 1g). First, PDA was synthesized via alkali‐induced pre‐polymerization, whereby colorless DA molecules in solution were oxidized by oxygen to quinone and immediately reacted with catechol to form black PDA.^[^ ^27^ ^]^ Subsequently, acrylamide monomers (AM), ammonium persulfate initiator (APS) initiators, and BIS crosslinkers were mixed with PDA and rapidly transformed into rGB/QCS/PDA–PAM hydrogel through in situ radical polymerization within 2 min. The chemical structure of rGB/QCS/PDA–PAM hydrogel is detailed in Figure 1g. The PAM network can maintain the integral elasticity of hydrogel and prevent deformations under external stress. Owing to the catechol/quinone chemical equilibrium of PDA, the internal PDA could act as a bridge to anchor with the flexible PAM network, rGB sheet and QCS with multiple irreversible and reversible covalent bonds and noncovalent interactions.^[^ ^28^ ^]^ The microscopic structure of the hydrogel was evaluated using SEM, which demonstrated that the nanosized rGB particles were well dispersed in the hydrogel and that small pores were intertwined with large pores (pore size 14.81 ± 6.8 and 36.38 ± 10.3 µm, respectively) to form a crosslinked network structure (Figure S3a, Supporting Information).

Moreover, swelling kinetics curves confirmed that the rGB/QCS/PDA–PAM hydrogel exhibited high water absorbability with ≈544% water absorption after a 30 h incubation (Figure S3b, Supporting Information), the swelling ratio was comparable with the previous reported hydrogel,^[^ ^29^ ^]^ indicating that the rGB/QCS/PDA–PAM hydrogel could exudate tissue secretions when used as wound dressing. Notably, swelling of rGB/QCS/PDA–PAM hydrogel induced some reduction in mechanical properties. After 2 h swelling of hydrogel, tensile stress decreased from 96.38 to 45.39 kPa, and tensile strain declined from 2630% to 1976% (Figure S8a,b, Supporting Information). The reason might be due to that the swelling decreased the solid content of the hydrogel and increased the molecular distance, thereby resulting in weakened non‐covalent interactions in the hydrogel network including hydrogen bond and *π*–*π* stacking interactions. Although there was a trade‐off between swelling ratio and mechanical performances of rGB/QCS/PDA–PAM hydrogel, the hydrogel still could meet the demand of application it as wound dressings in physiological environment.

To characterize the covalent and noncovalent interactions in the rGB/QCS/PDA–PAM hydrogel network, FT‐IR and XPS spectra were obtained. In FT‐IR spectra (Figure S3c, Supporting Information), compared with the pure PAM hydrogel, the new peak at 1258 cm^−1^ in the rGB/QCS/PDA–PAM hydrogel was attributed to the characteristic C—N stretching of phenol–amine interactions among the phenolic hydroxyl groups of PDA and —NH_2_ of PAM and QCS.^[^ ^30^ ^]^ The peaks at 3230 cm^−1^ ascribed to —OH vibration in the rGB/QCS/PDA–PAM hydrogel shifted to a higher wavenumber compared with those in other groups, indicating that hydrogen bonds had formed between the phenolic hydroxyl groups in PDA and the hydroxyl groups in QCS. In addition, the peak at 802 cm^−1^ corresponded to the B—O stretching vibration of boronic ester, which formed between the catechol group in PDA and the PBA group in rGB. Moreover, the interactions among these components in rGB/QCS/PDA–PAM hydrogel were further evaluated using XPS analysis (Figure 1h). The wide‐scan spectrum of rGB/QCS/PDA–PAM hydrogel displayed that the main elements of the hydrogel included C, N, O, and B. The deconvolution of C1s at 248.8, 268.5 and 288.1 eV were attributed to C—C, C=C, C—B bond, and C—N, C—O bond as well as NH_2_—C=O bond, respectively, confirming the presence of C—N interactions in the hydrogel (Figure 1i).^[^ ^31^ ^]^ In the deconvoluted B1s spectra, three typical peaks of B1s at 189.9, 190.2 and 191.7 eV were assigned to B—C, B—O—C and B—OH, respectively, implying the formation of dynamic boronic ester bonds derived from PDA and rGB in the hydrogel (Figure 1j).^[^ ^32^ ^]^ These multiple covalent and non‐covalent crosslinked networks of hydrogel could provide adaptability for treating dynamic wounds with frequent movement.

### 2.3 Mechanical Property and Self‐Healing Performance

**Figure 2:**
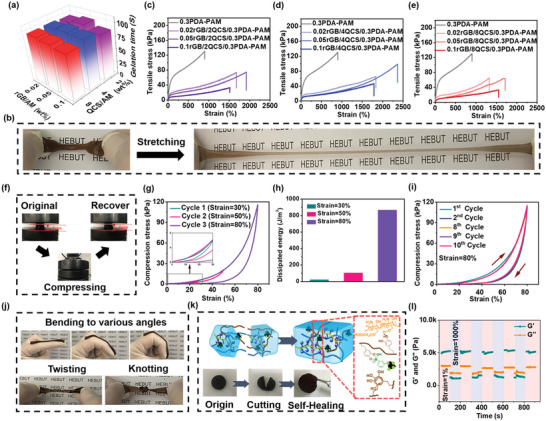
Mechanical property and self‐healing performance of rGB/QCS/PDA‐PAM hydrogel. a) Gelation time of hydrogels. b) Photos of the stretched rGB/QCS/PDA‐PAM hydrogel. c–e) Stress–strain curves of various rGB/QCS/PDA‐PAM hydrogel with different compositions (*n* = 3). f) Compressed photos of the optimized rGB/QCS/PDA‐PAM hydrogel. g) Sequential loading–unloading compression tests without interval of hydrogel under different strains (30%, 50%, and 80%), and the corresponding calculated h) dissipated energy. i) Recovered mechanical stability after compression tests for 10 successive loading–unloading cycles within 80% strain. j) Photos of the bending, knotting, and twisting of rGB/QCS/PDA‐PAM hydrogel. k) Self‐healing photos and mechanism. l) Step‐strain rheological for self‐healing.

The gelation time of hydrogel dressing is an important parameter for rapid hemostasis in clinical wound management.^[^ ^33^ ^]^ Therefore, the gelation time of rGB/QCS/PDA–PAM hydrogel with different contents of QCS and rGB was studied. The DA content on gelation time was first optimized because of the anchor role of PDA in hydrogel (Table S1, Supporting Information). Increasing the molar ratio of DA/AM from 0.1 to 0.5 prolonged the gelation time from 40 to 210 s (Figure S4a, Supporting Information). Once the DA/AM ratio was increased to >0.5, the hydrogel could not form because excess DA retarded AM polymerization by consuming free radicals. Consequently, a DA/AM ratio of 0.3 was selected for the PDA–PAM hydrogel (Figure S4b, Supporting Information). Furthermore, when various contents of QCS and rGB were incorporated into the PDA–PAM system (Table S2, Supporting Information), there was a negligible difference in the gelation time of rGB/QCS/PDA–PAM hydrogels, with ≈90 s (**Figure** **2**a), implying the critical role of PDA in forming hydrogel.

Desirable mechanical properties and stability are urgently needed in hydrogel dressings. Hydrogel dressings on wound healing are frequently reported to have brittle mechanical properties during use on dynamic wounds, such as joints and groins, causing unnecessary tearing‐induced wound infection and considerably shortened lifespan.^[^ ^34^ ^]^ Owing to the crosslinked network with multiple phenol–amine covalent bonds, reversible dynamic borate ester covalent bonds and noncovalent hydrogen bonds, the rGB/QCS/PDA–PAM hydrogel exhibited a high stretchability of up to tenfold the original length (Figure 2b). Stress–strain curves of rGB/QCS/PDA–PAM hydrogels with different QCS and rGB were studied (Figure 2c–e). Compared with the PDA–PAM hydrogel with low stretchability (902%), all rGB/QCS/PDA–PAM hydrogels exhibited increased stretchability ranging from 1327% to 2363%. This may be because the introduction of QCS and rGB enriched the noncovalent crosslinking network and the entanglement of macromolecular chains, effectively dissipating the energy when stretching. The 0.05rGB/4QCS/0.3PDA–PAM hydrogel attained the strongest tensile stress (98.03 kPa), highest tensile strain (2363%), most robust toughness (944.43 J m^−2^), and similar tensile modulus (4.031 kPa) in comparison with that of human skin (1–10 kPa) (Figure S5a–d, Supporting Information). Therefore, 0.05rGB/4QCS/0.3PDA–PAM was chosen for the following studies.

Self‐recovery and fatigue‐resistance ability are important for sustaining the lifespan of hydrogel; therefore, uniaxial cyclic compressive experiments were performed. A cylindrical specimen of rGB/QCS/PDA–PAM hydrogel could be completely compressed and immediately recovered after removing the force (Figure 2f). Further compression stress–strain curves with various successive compressive strain cycles were performed. As shown in Figure 2h, when compressed with no interval with strains from 30% to 80%, the hydrogel quickly returned to its original state after the load was released (Figure 2g), suggesting favorable fatigue resistance of the hydrogel. The compressive stress–strain curves manifested pronounced hysteresis loops, indicating ideal energy dissipation of the hydrogel. The dissipation energy for 30%, 50% and 80% of strain was 20.29, 103.83 and 865.24 J m^−3^, respectively (Figure 2h), indicating a typical elastic‐like behavior.^[^ ^35^ ^]^ The mechanical stability of the rGB/QCS/PDA–PAM hydrogel was tested using 10 consecutive loading and unloading cycles with a strain of 80%, which corresponded with the testing of hydrogel dressings here. As shown in Figure 2i, there were no changes of maximal compressive after 10 cycles, illustrating favorable mechanical stability of the rGB/QCS/PDA–PAM hydrogel. The flexibility, resilience and stability of the rGB/QCS/PDA–PAM hydrogel can be attributed to the multiple noncovalent hydrogen bonds provided by PDA, QCS and rGB, and reversible dynamic borate ester covalent bonds supplied by PDA and rGB. Thus, robust mechanical properties of the rGB/QCS/PDA–PAM hydrogel allowed it to be bent into various angles, with twisting and even knotting, which fulfills the requirement of wound dressings in dynamic human skin locations with high frequency movements (Figure 2j).

Notably, the noncovalent hydrogen bond of PDA with QCS and PAM, high dynamic reversible boronic acid esters covalent bonds of PDA with rGB, and *π*–*π* stacking of PDA may endow the rGB/QCS/PDA–PAM hydrogel with a favorable self‐healing ability as integrity can be recovered after experiencing stress caused by external forces.^[^ ^36^ ^]^ As the original circular hydrogel was cut into two semicircle pieces, these two patches could be put together and self‐healed into an integral hydrogel disk within 1 h (Figure 2k). To study the self‐healing properties of the hydrogel using rheological analysis, the linear viscoelastic region and gel–sol transition point of the hydrogel was checked using the strain sweep curve. This showed that the G′ and G″ values remained almost unchanged across a strain range of 0–42%, known as the linear viscoelastic region. With increasing strain, a sudden decrease in the G′ and G″ values occurred depicting gel‐to‐sol phase transition, and the two values intersected at a critical strain of 598% (Figure S6, Supporting Information). Therefore, a step‐strain rheological test to evaluate the self‐healing ability of hydrogel was conducted on the hydrogel by the repeat application of a larger strain of 1000% (>598%) and a smaller strain of 1% in the linear viscoelastic region. As shown in Figure 2l, upon applying the small strain, the G′ value exceeded that of G″, indicating a preserved gel state. However, when switching to the larger strain, both G′ and G″ values decreased and that of G′ suddenly appeared lower than that of G″, suggesting the collapse of hydrogel. When the strain returned to 1% again, both the G′ and G″ values recovered instantly with a G′ value exceeding that of G″, indicating the excellent self‐healing ability of rGB/QCS/PDA–PAM hydrogel. Therefore, the self‐healing properties of this hydrogel could maintain its integrity as wound dressing when countered with external deformations under dynamic movements.

### 2.4 Adhesive Properties

**Figure 3:**
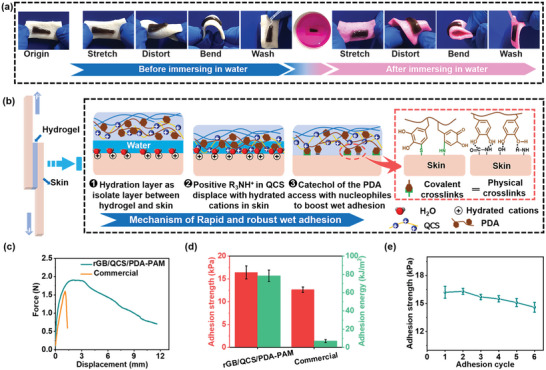
Adhesive properties of the rGB/QCS/PDA‐PAM hydrogel. a) Photographs of hydrogel adhered on porcine skin under different types of external deformations before and after water immersing. b) Schematic illustration of lap‐shear testing and proposed adhesive mechanisms. c) Force–displacement curves of rGB/QCS/PDA‐PAM hydrogel and commercial fibrin sealant on porcine skin, and the corresponding d) adhesive strength and adhesion energy. e) Adhesion stability of hydrogel after six peeling–adhesion cycles on porcine skin. Data are presented as mean ± SDs (*n* = 3).

Traditional hydrogel dressings often require gauze or suturing onto the wound region, and these external fixations can cause tight shielding on dynamic wounds and easily produce gaps for serious wound infection. Notably, recently developed adhesive hydrogel dressings could overcome these problems by tight adhesion and close coverage onto the wound surface as a physical barrier to prevent excessive blood loss and protect the wound region from infection.^[^ ^37^ ^]^ The rGB/QCS/PDA–PAM hydrogel demonstrated favorable adhesion to various types of dry substrates in ambient environment, including glass, rubber, alloy, stone, plastic and even could bear a 545 g weight (Figure S7a, Supporting Information). Further, the hydrogel showed excellent wet‐adhesion ability without any assistance on various tissues, such as heart, liver, spleen, lung, kidney and skin (Figure S7b, Supporting Information). To study the adhesion ability of hydrogel on dynamic movement skin with high movement frequency, a series of external stresses, including stretching, distorting and bending, were introduced before and after immersing in water for washing. Throughout these manipulations, hydrogel remained firmly adhered to the porcine skin without any separation (**Figure** **3**a and Movie S1, Supporting Information). These results indicated that the rGB/QCS/PDA–PAM hydrogel merited high adhesion ability and favorable skin adaptability. The outstanding wet adhesion mechanism of rGB/QCS/PDA–PAM hydrogel was proposed in Figure 3b. In wet tissue, the hydration layer present on the surface of tissue acted as isolation, which prevented the direct molecular interaction of catechol in the rGB/QCS/PDA–PAM hydrogel with nucleophiles in the tissue. The positive QAS R_3_NH^+^ group in the QCS of hydrogel could disrupt the hydrated isolate layer, displace hydrated cations on the surface of tissue, and enable the catechol of the PDA to access the nucleophiles (amines, thiol, and amide bond) on the surface of the wet tissue to form tight junctions via Michael addition and Schiff‐base reaction, causing the strong wet adhesion required for the synergistic interaction between the PDA and QCS on the hydrogel.^[^ ^38^ ^]^ Force–displacement curves were employed to quantitatively assess the adhesion strength of hydrogel on porcine skin, and commercial fibrin sealant was used as the Control group (Figure 3c). The maximum adhesion strength of hydrogel on fresh porcine skin (16.3 kPa) was slightly higher than that of commercial fibrin (12.68 kPa). The adhesion energy of the rGB/QCS/PDA–PAM hydrogel was 78.5 kJ m^−2^, which was 11.2‐fold higher than commercial fibrin glue (Figure 3d). Furthermore, rGB/QCS/PDA–PAM hydrogel also showed good adhesive strength and adhesive energy after immersing in PBS for 2 h, those value was 9.3 kPa and 21.815 kJ m^−2^, respectively (Figure S8c,d, Supporting Information), which was still higher than that of commercial fibrin sealant (12.68 kPa and 7.015 kJ m^−2^). Moreover, the rGB/QCS/PDA–PAM hydrogel could maintain excellent adhesion stability with only a minor decrease in adhesiveness after six cycles of peeling–adhesion tests, suggesting repeating and persistent adhesion stability (Figure 3e). Hence, the strong adhesive ability of the rGB/QCS/PDA–PAM hydrogel could effectively fix and integrate wound tissue, holding considerable potential for application in dynamic wounds.

### 2.5 NIR Triggered Photothermal Performance

**Figure 4:**
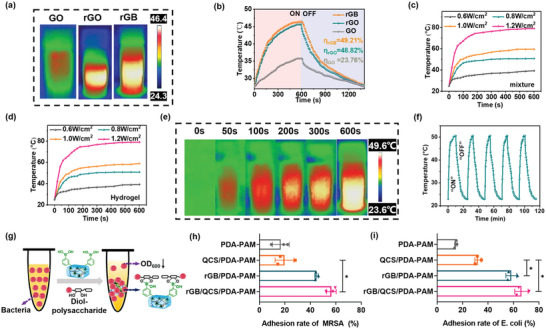
NIR responsive photothermal effects and of rGB/QCS/PDA‐PAM hydrogel. a) Thermal images and b) photothermal–conversion efficiency of GO, rGO, and rGB. Photothermal heating curves of c) rGB/QCS/PDA/AM mixture and d) rGB/QCS/PDA‐PAM hydrogel under different NIR irradiation powers for 10 min. e) Thermal images of rGB/QCS/PDA‐PAM hydrogel with mild irradiation powers (0.8 W cm^−2^). f) Photothermal stability of rGB/QCS/PDA‐PAM hydrogel with five on/off cycles. g) Bacterial capture via strong interactions between PBA on hydrogel and diol‐polysaccharide on bacterial wall. h,i) The adhesion rate of hydrogel on MRSA and *E. coli*. Data are presented as mean ± SDs (*n* = 3), *p < 0.05, Student's *t*‐test.

Initially, as there was a negligible temperature rise after 808‐nm irradiation for 600 s in the PDA–PAM hydrogel compared with that in the PAM hydrogel, the photothermal effect of PDA in the hydrogel was excluded (Figure S9a, Supporting Information). Before incorporation into hydrogel, the photothermal conversion efficiency of rGB sheets was evaluated using IR thermography. Under NIR irradiation at 808 nm for 10 min, the rGB solution showed a higher temperature compared with that of the rGO group and substantially higher than that of the GO group (**Figure** **4**a). Meanwhile, the quantitative photothermal efficiency (*η*) calculated by warming/cooling curves showed that the *η* of rGB (49.21%) was comparable with that of rGO (48.82%) and was considerably higher than that of GO (23.76%) (Figure 4b and Figure S9b–d, Supporting Information). These results indicated that rGB with PBA modification retained the excellent photothermal effect of rGO. The temperature changes of rGB aqueous solutions at different densities were monitored under NIR at 808 nm for 10 min. As shown in Figure 4c, upon NIR irradiation, the temperature of rGB aqueous solution continuously increased and was highly dependent on laser power density. Meanwhile, the photothermal effect of rGB/QCS/PDA–PAM hydrogel depicted similar power density dependent manner (Figure 4d), indicating no change in photothermal performance of rGB after incorporation into hydrogel.

Notably, the rGB/QCS/PDA–PAM hydrogel could reach 49.6 °C in 600 s at 0.8 W cm^−2^ power density (Figure 4e). This temperature was lower than 50 °C, meeting the basic biosafety requirements of mild antibacterial photothermal biomaterials.^[^ ^39^ ^]^ Furthermore, the photothermal effect of the rGB/QCS/PDA–PAM hydrogel could be switched on/off by NIR laser loading/unloading. The raised temperature was fully retained though five on/off laser cycles, depicting the excellent photothermal stability of rGB/QCS/PDA–PAM hydrogel (Figure 4f). Collectively, the rGB/QCS/PDA–PAM hydrogel with time–power multiple tunable mode, broad photothermal temperature ranges, and favorable photothermal stability shows a high potential to be used as NIR‐controlled photothermal antibacterial therapy.

### 2.6 Bacterial Capture Ability

Based on the glycocalyx‐mimicking characteristics of PBA, the rGB/QCS/PDA–PAM hydrogel was designed to capture bacteria via specific interactions between PBA and diol‐polysaccharide (lipopolysaccharide [LPS] in Gram‐negative bacteria and peptidoglycan [PGN] in Gram‐positive bacteria) on bacterial cell walls (Figure 4g). The binding affinities of hydrogel to Gram‐positive and Gram‐negative bacteria were, respectively, assessed by incubating hydrogels with MRSA and *E. coli* for 1 h and monitoring the solution turbidity at OD_600_. The adhesion rate was calculated using [1 − OD_600_ (hydrogel)/OD_600_ (PBS)] × 100%. As shown in Figure 4h, compared with the PDA–PAM hydrogel and QCS/PDA–PAM hydrogel without rGB, rGB/PDA–PAM, and rGB/QCS/PDA–PAM hydrogels exhibited an ≈4.38‐ and 2.77‐fold increased adhesion rate against with MRSA, respectively, indicating favorable bacterial capture capability of the rGB/QCS/PDA–PAM hydrogel on Gram‐positive bacteria. The rGB/QCS/PDA–PAM hydrogel also displayed excellent bacterial adhesion capability for Gram‐negative bacteria. As shown in Figure 4i, the adhesion rate of the rGB/QCS/PDA–PAM hydrogel on *E. coli* was comparable with that of the rGB/PDA–PAM hydrogel, which was 4.44‐ and 2.58‐fold higher than that of the PDA–PAM and QCS/PDA–PAM hydrogel, respectively, indicating the bacterial capture capability of the rGB/QCS/PDA–PAM hydrogel achieved by decorating with PBA. The strong bacterial capture ability of hydrogel would act as efficient antibacterial therapy.

### 2.7 Mild PTT Antibacterial Effect and Mechanism

**Figure 5:**
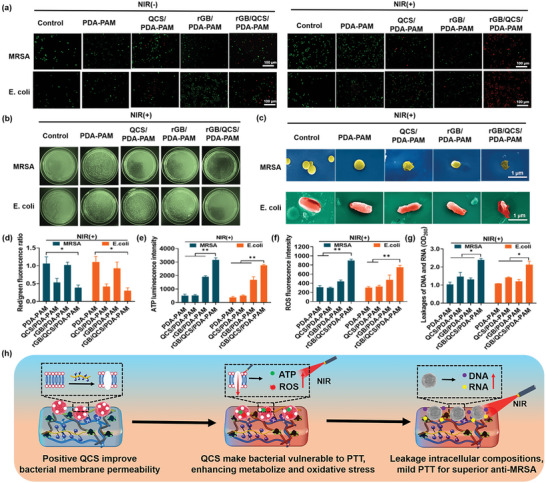
Antibacterial activity and mechanism of rGB/QCS/PDA‐PAM hydrogel. a) Fluorescence images of the live/dead (green/red) bacteria treatment via different hydrogel with or without mild NIR irradiation (0.8 W cm^−2^, 600 s). b) Optical images of MRSA and *E. coli* bacterial colonies after different treatments under mild NIR irradiation (0.8 W cm^−2^, 600 s). c) SEM image of MRSA and *E. coli*. The changes of MRSA and *E. coli* after treatment with different hydrogel under mild NIR irradiation (0.8 W cm^−2^, 600 s), including d) bacterial membrane potentials, e) ATP level, f) ROS level and g) DNA and RNA leakage. h) Schematic illustration of the antibacterial mechanism of the rGB/QCS/PDA‐PAM hydrogel. Data are presented as mean ± SDs (*n* = 3), *p < 0.05, ^**^ *p* < 0.01 Student's *t*‐test.

As the temperature of mild PTT antibacterial activity requires a temperature of <50 °C, the NIR laser irradiation at 808 nm at 0.8 W cm^−2^ for 600 s was chosen to ensure the raising temperature retained to 49.6 °C (<50 °C). The antibacterial effect of rGB/QCS/PDA–PAM hydrogel was investigated by coincubating with Gram‐positive MRSA and Gram‐negative *E. coli*. The antibacterial activity was assessed using live/dead staining, where live bacteria exhibit green fluorescence and dead bacteria exhibit red fluorescence. As shown in **Figure** **5**a, without NIR irradiation, the PDA–PAM hydrogel and rGB/PDA–PAM hydrogel exclusively exhibit green fluorescence, suggesting negligible antimicrobial effect on MRSA and *E. coli* without photothermal production. The QCS/PDA–PAM and rGB/QCS/PDA–PAM hydrogels appeared to display comparable red florescence, indicating that the antibacterial role of rGB/QCS/PDA–PAM hydrogel without NIR laser was dominated by QCS. Upon NIR irradiation, the QCS/PDA–PAM hydrogel exhibited comparable red fluorescence as the hydrogel without NIR laser, whereas the rGB/PDA–PAM hydrogel exhibited partial red fluorescence owing to the mild temperature increase after NIR irradiation, and rGB/QCS/PDA–PAM hydrogel showed almost entirely red fluorescence caused by the synergistic antibacterial action of QCS and rGB. These results implied that only with the cooperation of QCS with rGB in hydrogel, NIR‐triggered efficient mild PTT antibacterial at low temperatures (49.6 °C) could be realized. Subsequently, the excellent mild PTT antibacterial effect was also confirmed by plate counting (Figure 5b). The quantitative antibacterial efficiency of PDA–PAM, QCS/PDA–PAM, rGB/PDA–PAM and rGB/QCS/PDA–PAM hydrogels against MRSA was 8.8%, 24.4%, 18.5% and 94.6%, respectively, and against *E. coli* was 6.8%, 23.6%, 11.4% and 96.6% (Figure S10, Supporting Information), respectively. Notably, the comparable antibacterial effect for previously reported photothermal antibacterial materials required a photothermal temperature of up to 72.8 °C, which was considerably higher than physiological temperature (37 °C) and often induced serious irreversible thermal injury to normal tissue. Hence, our rGB/QCS/PDA–PAM hydrogel exhibited mild antibacterial activity with high efficiency, guaranteeing acceptable biosafety for application in MRSA‐infected wound management.

To further explore the antibacterial mechanism of the rGB/QCS/PDA–PAM hydrogel, a series of experiments were performed. First, microscopic morphology of bacteria after different treatments was observed using SEM (Figure 5c and Figure S11, Supporting Information). Upon NIR laser irradiation, after PDA–PAM and rGB/PDA–PAM hydrogel treatment, both MRSA and *E. coil* preserved an integrated cell membrane with a smooth surface, whereas with QCS/PDA–PAM hydrogel treatment, clearly wrinkled and porous cell membranes were observed. Conversely, the rGB/QCS/PDA–PAM group exhibited the most considerable large lesions and atrophy, and the dead bacteria wound drop off from the surface of hydrogel (Figure S12, Supporting Information). The explanation might be that lipopolysaccharide (LPS) in Gram‐negative bacteria and peptidoglycan (PGN) in Gram‐positive bacteria was released when the bacteria were killed,^[^ ^40^ ^]^ the specific interactions between PBA in rGB/QAS/PDA‐PAM hydrogel and diol‐polysaccharide on bacterial cell walls would be disrupted. Quantitative membrane potential changes of bacteria after various treatments were detected using the 3,3‐diethyloxacarbocyanine probe (DiOC2(3)). This probe emits red fluorescence in healthy bacteria with high membrane potential and shifts to green fluorescence in disrupted membranes with low potential, and the red/green fluorescence ratio is usually employed to evaluate the degree of the bacterial membrane damage.^[^ ^41^ ^]^ As shown in Figure 5d, under NIR laser irradiation, the lowest red/green value was found in the rGB/QCS/PDA–PAM group and was particularly lower than that of the rGB/PDA–PAM group. The damage to the bacterial membrane and decrease in membrane potential suggested that QCS disrupted the integrity of the bacterial membrane and that the damage could be exacerbated by mild PTT of rGB in the hydrogel. Furthermore, as bacterial metabolism was closely correlated with intracellular temperature, the level of adenosine triphosphate (ATP), a common indictor for bacterial metabolism, was tested after bacterial treatment using hydrogel with NIR.^[^ ^42^ ^]^ Compared with that of the other hydrogel treatments, the ATP fluorescence was considerably enhanced in the rGB/QCS/PDA–PAM group (Figure 5e). Because hyperthermia‐accelerated metabolism could promote a series of single‐electron reduction and thereafter induce reactive oxygen species (ROS) production, the intracellular ROS in MRSA and *E. coli* after different treatments were stained using the DCFH‐DA probe.^[^ ^43^ ^]^ As demonstrated in Figure 5f, consistent with the ATP results, rGB/QCS/PDA–PAM showed the strongest ROS fluorescence intensity. In particular, the bacterial metabolism (ATP and ROS) of the rGB/QCS/PDA–PAM group was considerably stronger than that in the rGB/PDA–PAM group and was likely caused by the temperature increase resulting from the enhancement of NIR light permeability induced by QCS damage to the bacterial membrane. Finally, the leakage of DNA and RNA after bacterial death was assessed using an OD_260_ assay (Figure 5g).^[^ ^44^ ^]^ The rGB/QCS/PDA–PAM group with the highest value of OD_260_ displayed the most leakage of abundant cellular components (DNA and RNA), indicating the prominent antibacterial effect of the rGB/QCS/PDA–PAM hydrogel.

The antibacterial mechanism of the rGB/QCS/PDA–PAM hydrogel is summarized in Figure 5h. Positive QCS punctured the negative cell membrane by electrostatic disruption in a contact pattern for improved membrane permeability. Upon NIR irradiation, the QCS‐improved NIR permeability made the bacteria highly susceptible to the photothermal effect by promoting bacterial metabolism (ATP) and elevating intracellular oxidative stress (ROS), thus causing bacterial death and leakage of abundant intracellular compositions (DNA and RNA). Therefore, the rGB/QCS/PDA–PAM hydrogel could achieve a mild photothermal antibacterial (M‐PTT) effect with high efficiency at low temperature.

### 2.8 Cytocompatibility and Hemocompatibility

**Figure 6:**
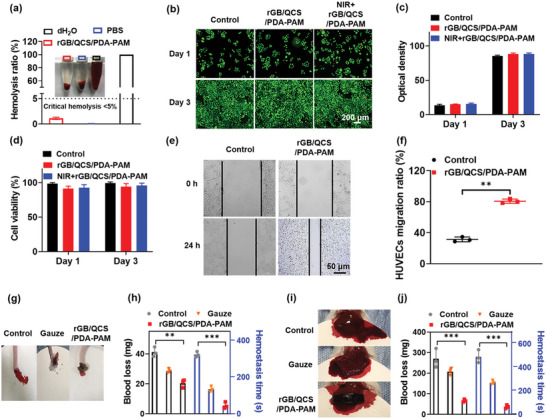
The biosafety, cells migration, and hemostatic capability of rGB/QCS/PDA‐PAM hydrogel. a) Hemolysis test of the rGB/QCS/PDA‐PAM hydrogel, compared to positive control (dH_2_O) and negative control (PBS). b) Live/Dead (green/red) fluorescence images of L929 cells after incubation with NIR+rGB/QCS/PDA‐PAM hydrogel, rGB/QCS/PDA‐PAM hydrogel and PBS for 1 and 3 days, and c) quantitative fluorescence intensity of live L929 cells. d) Cell viability of L929 cells after NIR+rGB/QCS/PDA‐PAM hydrogel, rGB/QCS/PDA‐PAM hydrogel, and PBS treatment. e,f) Optical images and the migration ratio of HUVECs after rGB/QCS/PDA‐PAM hydrogel and PBS treatment for 24 h. g–j) Hemostasis images and quantification data of Control, gauze, and rGB/QCS/PDA‐PAM hydrogel group on mouse‐tail amputation model and mouse‐liver hemorrhage model; Data are presented as mean ± SDs (*n* = 3), *p < 0.05, **p < 0.01, ***p < 0.001, Student's *t*‐test.

Biosafety, including hemocompatibility and cytocompatibility, is a prerequisite for wound dressing applications.^[^ ^45^ ^]^ The hemocompatibility of hydrogels was evaluated via hemolysis tests. As shown in **Figure** **6**a, the positive control (distilled water) exhibited strong hemolysis, whereas the negative control (PBS) and rGB/QCS/PDA–PAM hydrogel exhibited no hemolysis. The hemolysis rate of positive control, PBS and rGB/QCS/PDA–PAM hydrogel on blood red cells was 100%, 0.1% and 1.14 ± 0.22%, respectively, indicating the favorable hemocompatibility of the rGB/QCS/PDA–PAM hydrogel. Moreover, the cytocompatibility of rGB/QCS/PDA–PAM was evaluated by incubating this with L929 fibroblast cells and staining cells using the Live/Dead kit. For all groups, the green fluorescence representing live cells gradually increased because of cell proliferation after culturing for 1 and 3 d (Figure 6b), and there was no difference in quantitative fluorescence intensity among these groups (Figure 6c). Notably, there was a high cell viability with the NIR + rGB/QCS/PDA–PAM hydrogel, which proved that the mild photothermal temperature did not harm cell growth or survival. As in a previous report, when the cell is exposed to environmental temperatures >50 °C, cell damage will rapidly result from protein degeneration and DNA degradation.^[^ ^46^ ^]^ Furthermore, the cell viability of the irradiation and control groups was prolonged for 1 and 3 d (Figure 6d), confirming the favorable cytocompatibility of the rGB/QCS/PDA–PAM hydrogel with mild photothermal effect.

### 2.9 Cell Migration and Hemostatic Capacity

Previous studies reported that hydrogels with a 3D porous structure could provide an extracellular matrix‐mimicking environment to promote cell migration.^[^ ^47^ ^]^ Therefore, cell migration on the rGB/QCS/PDA–PAM hydrogel was evaluated. As shown in Figure 6e, rGB/QCS/PDA–PAM hydrogel considerably promoted the migration of HUVECs, as indicated by the higher migration ratio (80.86%) compared with that of the Control group (31.34%) (Figure 6f). Additionally, the migration ratio was improved by the hydrogel in L929 cells (Figure S13a,b, Supporting Information). These results suggest that the rGB/QCS/PDA–PAM hydrogel could promote the migration of endothelial cells and fibroblasts and was conducive to wound healing when used as wound dressing.

Hemostatic performance is the fundamental demand for the wound dressing in the early stages of wound healing.^[^ ^48^ ^]^ To evaluate the pro‐coagulant rate of the hydrogels in vitro, the coagulation time of whole blood and blood clotting index (BCI) were first determined (Figure S14a,b, Supporting Information). Compared to blank group (576 s) and Gauze (460 s), rGB/QCS/PDA‐PAM hydrogel group showed the shortest clotting time (277 s), indicating the pro‐coagulant effect of the hydrogels. Furthermore, BCI of rGB/QCS/PDA‐PAM hydrogel group (31.34%) was also significantly lower than Gauze group (63.33%), suggesting a higher rate of blood clot formation. To explore the mechanism of the hemostasis, the attachment of platelet on the surface of hydrogel was determined using LDH kits, which was the typical blood coagulation regimes. Results exhibited that Attachment of platelet of rGB/QCS/PDA‐PAM hydrogel group (44.67%) was obviously higher than Gauze group (19.88%), which would contribute to the good procoagulant effect (Figure S14c, Supporting Information). These results implied that rGB/QCS/PDA‐PAM hydrogel merited good hemostatic performance in vitro. To evaluate the hemostatic property of the rGB/QCS/PDA‐PAM hydrogel in vivo, acute massive bleed model with mouse‐tail amputation (exter‐hemorrhage) and mouse‐liver hemorrhage (inter‐hemorrhage) were established. And the wounds were then treated with rGB/QCS/PDA‐PAM hydrogel, Gauze, and blank, respectively. For both mouse‐tail amputation (Movies S2 and S3, Supporting Information) and mouse‐liver hemorrhage model (Movies S4 and S5, Supporting Information), extensive blood flow was found in PBS and Gauze group, while rGB/QCS/PDA‐PAM hydrogel treated group completely blocked excess bleeding (Figure 6g,i). Considering acute hemorrhage occurred within 100 s after wound, fast gelation was extreme required for wound dressing. Hemostasis time in mouse‐tail amputation significantly shortened, from 272 s in Gauze group to 46 s in rGB/QCS/PDA‐PAM group, and the blood loss decreased from 33 mg in Gauze group to 20 mg in rGB/QCS/PDA‐PAM group (Figure 6h). Similarly, hemostasis time in mouse‐liver hemorrhage model significantly shortened from 271 s in Gauze group to 46 s in rGB/QCS/PDA‐PAM group, and the blood loss decreased from 207 mg in Gauze group to 66 mg in rGB/QCS/PDA‐PAM group (Figure 6j). The quickly coagulation and minimize blood loss suggested the good hemostatic performance of rGB/QCS/PDA‐PAM hydrogel on various acute bleeding trauma. The excellent hemostatic performance and cell migration of rGB/QCS/PDAPAM hydrogel would diminish bacterial infection induced by bleeding at early stage and facilitate healing related cells infiltration at later wound healing.

### 2.10 Full‐Thickness MRSA‐Infected Skin Wound Healing

**Figure 7:**
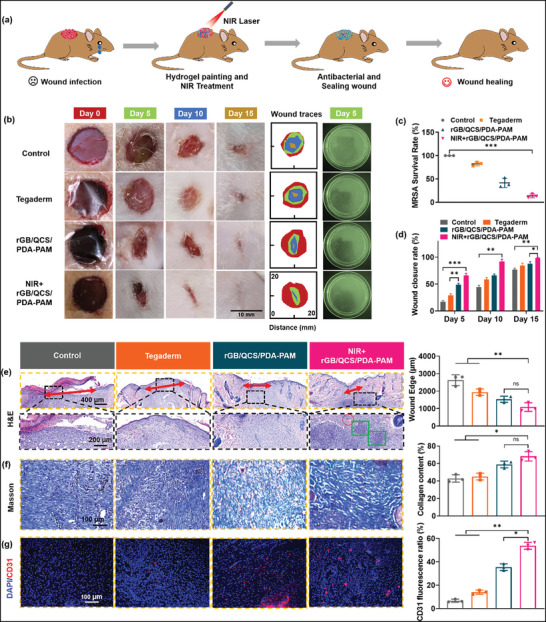
MRSA‐infected wounds healing in vivo. a) Treatment process of full‐thickness MRSA‐infected skin wound healing. b) Representative photos and wound traces after various treatments at day 0, 5, 10, 15, and bacterial colonies from wound of different treatment groups on day 5. c) Quantification data of antibacterial effect in vivo on day 5. d) Wound closure rates calculated from photo‐images (scale bar: 10 mm). e) Histopathology H&E staining and the calculated wound edges (red arrows) at day 15, red circle and green box represent hair follicles and nascent granulation. f) Masson's trichrome staining for collagen deposition at day 15. g) CD31 staining for vascular endothelial cells at day 15. Data are presented as mean ± SDs (*n* = 3), *p < 0.05, **p < 0.01 ***p < 0.001. Student's *t*‐test.

Dynamic wounds infected with drug‐resistant bacteria are often difficult to cure because of frequent wound movement and poor tissue regeneration. The rGB/QCS/PDA–PAM hydrogel can solve these problems. An MRSA‐infected full‐thickness skin defect model was constructed on the back of SD rats and treated with the rGB/QCS/PDA–PAM hydrogel (**Figure** **7**a).

To investigate the antibacterial effect of the hydrogel during the wound healing process in vivo, tissue fluids were collected from the wound surface after different treatments for 5 d. The extracted MRSA was incubated on agar plates, and the bacterial colonies were counted (Figure 7b). The results showed that almost no bacterial colonies were found for the NIR + rGB/QCS/PDA–PAM hydrogel, whereas many bacterial colonies were present in the other groups (Figure 7c), indicating the effective antibacterial effect of the rGB/QCS/PDA–PAM hydrogel with NIR irradiation in vivo. Moreover, during the 15‐d treatment period, the wound size progressively reduced from 0 to 15 d for all groups, with the NIR + rGB/QCS/PDA–PAM group demonstrating the fastest rate of wound healing. The wound closer rate after 15 d of treatment for the NIR + rGB/QCS/PDA–PAM group was almost 100% (Figure 7d), whereas those for the rGB/QCS/PDA–PAM, commercial Tegaderm, and Control groups were ≈87.4%, ≈83.5% and ≈77.1%, respectively, confirming the accelerated wound healing effect of NIR + rGB/QCS/PDA–PAM. Notably, negligible weight changes were found for SD rats during NIR + rGB/QCS/PDA–PAM hydrogel treatment, underlining the biosafety of hydrogel treatment (Figure S15, Supporting Information).

Further histopathology evaluations of regenerated skin after treatment for 15 d were assayed by H&E, Masson, and CD31 immunofluorescence staining. As shown in Figure 7e, at Day 15, an intact epidermis with a thin epithelial layer was observed in the NIR + rGB/QCS/PDA–PAM hydrogel group, indicating considerable wound healing. Conversely, thickened and inhomogeneous epidermis was present in the rGB/QCS/PDA–PAM hydrogel and Tegaderm groups, and an incomplete epidermis was present in the Control group. Furthermore, the average length of the wound edge for the NIR + rGB/QCS/PDA–PAM and rGB/QCS/PDA–PAM hydrogels and the Tegaderm, and Control groups was 1088.4, 1534.2, 1942.5 and 2626.8 µm, respectively. Moreover, the NIR + rGB/QCS/PDA–PAM hydrogel‐treated wounds showed the thinnest epithelial at Day 15 (Figure S16, Supporting Information). Additionally, compared with other treatment groups, dermis appendages, including hair follicles and granulation, were found in the NIR + rGB/QCS/PDA–PAM hydrogel group. Masson staining was employed to assess collagen deposition and arrangement of the healing skin (Figure 7f). Abundant collagen with dense and organized structures could be observed in the NIR + rGB/QCS/PDA–PAM hydrogel group, whereas the other groups contained fewer collagen fibers with a loosely packed distribution. The quantitative collagen content was analyzed by calculating the positively blue‐stained area. Compared with that in the Tegaderm and rGB/QCS/PDA–PAM hydrogel groups, a considerably higher content of collagen in the dermis was found in the NIR + rGB/QCS/PDA–PAM hydrogel group. Angiogenesis is an essential parameter for wound regeneration. CD31, a classical marker expressed in vascular endothelial cells, was evaluated by immunofluorescence staining. As shown in Figure 7g, marked red fluorescence was observed in the NIR + rGB/QCS/PDA–PAM group. The relative capillary intensity of the NIR + rGB/QCS/PDA–PAM group (52.38%) was considerably higher than those in the Control (7.15%), Tegaderm (13.20%) and rGB/QCS/PDA–PAM without NIR groups (36.87%), indicating the proangiogenic effect of the NIR + rGB/QCS/PDA–PAM hydrogel treatment. Overall, these results clearly demonstrated that the rGB/QCS/PDA–PAM hydrogel with NIR irradiation considerably promoted MRAS‐infected wound healing with an intact epidermis and regeneration of other appendages via abundant collagen deposition and prominent angiogenesis.

## 3 Conclusion

We successfully prepared a versatile hydrogel (rGB/QCS/PDA–PAM) dressing with tissue‐adaptable performance and efficient antibacterial activity for MRSA‐infected dynamic wound healing. The multiple covalent and noncovalent crosslinking network of rGB/QCS/PDA–PAM hydrogel endowed this with modest mechanical strength (tensile modulus of 4.031 kPa), strong tissue adhesiveness (adhesion energy of 78.5 KJ m^−2^), and favorable self‐healing performance to adapt to dynamic wounds. Moreover, a glycocalyx‐mimicking phenylboronic acid‐functioned rGB in the hydrogel could capture bacteria and enhance the bacterial effect of QCS by disrupting the bacterial membranes. Furthermore, as the incomplete bacterial membrane became vulnerable to photothermal effect, the hydrogel showed high antibacterial activity against MRSA at mild temperatures (49.6 °C) in vitro. The biocompatible hydrogel considerably accelerated the tissue regeneration of MRSA‐infected wound in vivo, with an intact epidermis and abundant collagen deposition as well as extensive angiogenesis. Overall, this multifunctional hydrogel was a promising candidate for clinical bacteria‐infected wound management.

## 4 Experimental Section

### Synthesis and Characterization of QCS and rGB

rGB was prepared by modification 3‐aminophenylboronic acid on the surface of rGO using previous reported method with some modifications.^[^ ^49^ ^]^ QCS were fabricated by conjugating QP on to CMCS at a nitrogen‐protected atmosphere.^[^ ^50^ ^]^ The details were available in the Experimental Methods of Supporting Information.

### Preparation and Characterization of rGB/QCS/PDA‐PAM Hydrogel

rGB/QCS/PDA‐PAM hydrogel was prepared through free radical polymerization. First, dopamine (DA) was pre‐polymerized in Tris‐HCl solution (pH = 8.5) for 20 min, the color changed from colorless and transparent to black‐brown, proving the successful pre‐polymerization.^[^ ^51^ ^]^ Subsequently, rGB, QCS, acrylamide monomer (AM), ammonium persulfate initiator (APS), cross‐linker (BIS) and tetramethyl‐ethylenediamine catalyzer (TMEDA) were added to the PDA solution, free radical polymerization was conducted at room temperature and rGB/QCS/PDA‐PAM hydrogel was obtained. The homogeneous prepolymer solutions obtained above were transferred various reaction models and kept for 10 min. The sample of 0.05rGB/4QCS/PDA‐PAM hydrogel represented that the weight ratios of QCS/AM and rGB/AM was 4 and 0.05, and the weight percent of DA/AM was fixed to be 0.3. XPS spectra, IR spectra, SEM was used to characterize the structure of hydrogel. Gelation time and Swelling ratio of hydrogel was tested, the details could be found in the Experimental Methods of Supporting Information.

### Mechanical Performance Tests

Mechanical test of the hydrogels was investigated by conducting the uniaxial tensile test and compressive test. For tensile tests, the hydrogels with various compositions were poured in a dumbbell‐shaped PTFE model. The stretchable section measure was 16 mm ×4 mm× 2 mm (length × width × thickness), and the crosshead speed set at 100 mm min^−1^. For compressive tests, hydrogels were prepared in a cylindrical shape with a diameter of 20 mm and a height of 8 mm, and the crosshead speed set at 10 mm min^−1^. Tensile modulus was obtained from the slope of the stress–strain curve within 5% strain. Sequential loading–unloading compression tests without interval of rGB/QCS/PDA‐PAM hydrogel under different strains (30%, 50% and 80%) were tested. In addition, recovered mechanical stability of rGB/QCS/PDA‐PAM hydrogel after compression tests for 10 successive loading unloading cycles within 80% strain was tested (*n* = 3).

### Self‐Healing Test

rGB/QCS/PDA‐PAM hydrogel with 20 mm diameter and 8 mm thickness was cut into two pieces, then they were allowed to self‐healing at room temperature. After 1 h incubation, self‐healing hydrogel was documented by photography. Meanwhile, the self‐healing behavior of the hydrogel was also investigated by alternate strain sweep test at a fixed angular frequency (6.28 rad s^−1^) under continuous train sweep with small strain (*γ* = 1.0%, 100 s for each interval) to large strain (*γ* = 1000%, 100 s for each interval).

### Adhesive Performance Tests

To study the adhesion ability of rGB/QCS/PDA‐PAM hydrogel in wet condition, hydrogel was immersed in PBS for 1 h, a series of external stresses was introduced before and after immersing in water, including stretching, distorting, bending and washing. Then, adhesive ability of the rGB/QCS/PDA‐PAM hydrogel on fresh porcine skin (bonding area, 10 × 8 mm) was evaluated by a lap‐shear test. Hydrogels were pulled via microcomputer controlled electronic universal testing machine at a crosshead speed of 20 mm min^−1^, until the two pieces separated. Adhesive strength was calculated as the maximum force divided by the bonding area. (*n* = 3)

### Photothermal Effect Measurements

GO, rGO and rGB was irradiated with NIR laser (808 nm, 1 W cm^−2^, 10 min), the heat distribution and temperature increase were determined by an infrared thermal imager, and the photothermal conversion efficiency of GO, rGO and rGB was calculated according previous reported method.^[^ ^52^ ^]^ Moreover, rGB sheet and rGB/QCS/PDA‐PAM hydrogel was irradiated with different powers of NIR energy (0.6, 0.8, 1, and 1.2 W cm^−2^) for 600 s to assess the time‐power multiple tunable mode. The photothermal stability of rGB/QCS/PDA‐PAM hydrogel was tested at 0.8 W cm^−2^ by turning on and off NIR laser.

### Bacterial Capture, Antibacterial Activity, and Mechanism

The bacterial capture of hydrogel towards MRSA and *E. coli* was evaluated by a turbidimetric method. Antibacterial activity and mechanism of hydrogels were determined by colonies forming unit assay (CFU), Live/Dead staining, leakage of DNA and RNA, ROS level, ATP level and membrane potential changes. The bacterial morphology after hydrogel treatment was observed by SEM. Details for these experiments are described in the Supporting Information.

### In Vitro Cytotoxicity, Cell Migration, Hemolysis, and Hemostasis tests

Cytotoxicity of rGB/QCS/PDA‐PAM hydrogel was evaluated on the L929 fibroblast cell by CCK‐8 kit and live/dead assay. Cell migration of HUVECs and L929 fibroblast cells on the hydrogel was carried out using typical scratch assay. Hemocompatibility of rGB/QCS/PDA‐PAM hydrogel was evaluated on red blood cells (RBCs) collected from rat's fresh blood by in vitro hemolysis assay. Hemostatic performance of the rGB/QCS/PDA‐PAM hydrogel was evaluated Blood Coagulation Test,^[^ ^53^ ^]^ BCI,^[^ ^54^ ^]^ Attachment of Platelets,^[^ ^55^ ^]^ mouse ‐tail amputation model and mouse‐liver hemorrhage model. Details for these experiments are described in the Supporting Information.

### In Vivo Healing of Full‐Thickness MRSA‐Infected Wounds Tests

Full‐thickness wounds of the MRSA‐infected full‐thickness round skin wounds models were established, accelerating wound healing performances of rGB/QCS/PDA‐PAM hydrogel was evaluated by wound closer rate. After 15‐days treatment, H&E staining, Masson's trichrome, and CD31 staining were implemented to assess the wound healing mechanism. Details for these experiments are described in the Supporting Information.

### Ethical Approval

Due to animal experiments included in the authors’ manuscript, ethical approval from Laboratory Animal Ethics Committee, Institute of Radiology, Chinese Academy of Medical Sciences was obtained prior to the research, and the assigned approval number was IRM‐DWLL‐2020061.

### Statistical Analysis

Data were presented as mean ± standard deviations (SDs). Student's *t*‐test or one‐way ANOVA was used to assess the difference between two groups or multiple groups using SPSS software, respectively. The levels of significance were labeled with *p < 0.05, **p < 0.01, and ***p < 0.001.

## Conflict of Interest

The authors declare no conflict of interest.

## Supporting information

Supporting Information

Supplemental Movie 1

Supplemental Movie 2

Supplemental Movie 3

Supplemental Movie 4

Supplemental Movie 5

## Data Availability

The data that support the findings of this study are available from the corresponding author upon reasonable request.
