# Supplementary material for: Versatile Hydrogel Dressing with Skin Adaptiveness and Mild Photothermal Antibacterial Activity for Methicillin‐Resistant Staphylococcus Aureus‐Infected Dynamic Wound Healing
Source: Adv Sci (Weinh). 2023 Feb 12;10(11):2206585. doi: 10.1002/advs.202206585 (PMC10104652; doi:10.1002/advs.202206585)
Supplement: Supplementary file 1 — Supporting Information [file ADVS-10-2206585-s004.pdf]

ADVANCED SCIENCE
Open Access

Supporting Information

for *Adv. Sci.*, DOI 10.1002/advs.202206585

Versatile Hydrogel Dressing with Skin Adaptiveness and Mild Photothermal Antibacterial Activity for Methicillin-Resistant Staphylococcus Aureus-Infected Dynamic Wound Healing

Peng Zhao, Yu Zhang, Xiaoai Chen, Chang Xu, Jingzhe Guo, Meigui Deng, Xiongwei Qu, Pingsheng Huang, Zujian Feng* and Jimin Zhang**

Supporting Information

Versatile hydrogel dressing with skin adaptiveness and mild photothermal antibacterial activity for Methicillin-resistant Staphylococcus aureus-infected dynamic wound healing

Peng Zhao, Yu Zhang, Xiaoai Chen, Xu Chang, Jingzhe Guo, Meigui Deng, Xiongwei Qu, Pingsheng Huang, Zujian Feng* and Jimin Zhang**

Experimental methods

Materials

Dopamine hydrochloride and Graphene oxide (GO, 5 mg/mL in water) were purchased from Anhui Energy Chemical Reagent Co., Ltd. (China) and Shanxi Tanmei Technology Reagent Co., Ltd. (China), respectively. Carboxymethyl chitosan (CMCS) (Degree of carboxymethylation=80%) was purchased from Jiangsu Feiyubio Reagent Co., Ltd. (China). 3-aminophenylboronic acid (PBA)and Ascorbic Acid (L-Ascorbic acid 2-phosphate sesquimagnesium salt hydrat) were purchased from Shanghai Bide Pharmatech Reagent Co., Ltd. (China) and Shanghai Haohong bio-medical Reagent Co., Ltd. (China), respectively. Isophorone diisocyanate (IPDI), Acrylamide (AM), ammonium persulfate (APS), N, N′ -methylene bisacrylamide (BIS), and N, N, N′, N′ - Tetramethyldiamine (TEMED) were received from the Aladdin Chemical Reagents Co., Ltd. (China). All chemical reagents were used without further purification. Balb/c mice (20 ± 2 g) or Sprague-Dawley (SD)rats (200± 20 g) were purchased from Beijing Vital River Laboratory Animal Technology Co., Ltd. (Beijing, China). Animals were housed an SPF-class laboratory and allowed free access to food and water. All animal care and experimental procedures were approved by Animal Experiment Ethics Committee and Authority of Institute of Radiation Medicine, Chinese Academy of Medical Sciences (Approval number: IRM-DWLL-2020061).

Characterization

Chemical structure was characterized using ^{1}H NMR spectroscopy (400 MHz; AVANCE 400, Bruker biospin AG, Swiss), FT-IR spectrometer (TENSOR 27, BRUKER, Germany), UV–vis spectrophotometer (CARY300, Varian, USA), Raman spectroscopy (Takram P50C0R10, Renishaw, Iran). Zeta potential was measured by Zetasizer Nano-ZS90 system (Malvern Instruments, Worcestershire, England). Morphology of hydrogel was investigated by scanning electron microscope (SEM, Nova Nano SEM450, FEI, USA). The hydrogel samples were lyophilized by a freeze drier (FD-1A-50, Boyikang, China). Based on the SEM images, the pore size distribution of hydrogel was counted through Image J software. The rheology properties of hydrogels were evaluated by an TA rheometer (AR 2000ex, TA, USA). Uniaxial

tensile and compressive test using electronic universal testing machine (CMT6104, Xinsansi, China). Photothermal effect was monitored with a FLIR I5 infrared thermal camera (I5, FLIR, USA).

Synthesis of 3-aminophenylboronic acid functionalized reduced graphene oxide (rGB)

GO solution (5 mg/mL, 2 mL) and deionized water (18 mL) were put in a 50 mL glass vial equipped with a cooling reflux unit, 3-aminophenylboronic acid (PBA, 30 mg) was added and the reaction mixture was vigorously stirred at 70°C for 3 h. Then, 20 mg ascorbic acid were added and the temperature was raised to 80 °C with constant stirring for 1 h. The crude product was washed thoroughly with water to remove unreacted PBA. Precipitation and redispersion was conducted for more than three times, rGB was obtained and finally stored in water. IR spectra, UV–vis absorption spectra, Raman spectra was used to characterize the chemical structure of rGB.

Synthesis of quaternary ammonium carboxymethyl chitosan (QCS)

5-Phenyl-1,3,4-oxadiazole quaternary ammonium salts (QP, 0.5 g) and isophorone diisocyanate (IPDI, 0.208 g) with molar ratio 1.05:1 were reacted in dimethylformamide (DMF, 10 mL) at 25 °C for 3 h. Subsequently, the solution was precipitated in cold diethyl ether. The precipitate was filtered and then dried in a vacuum oven to obtain a yellowish powder (QP-IPDI conjugate). Then, QP-IPDI conjugate reacted with CMCS at different molar ratio (0.2:1, 0.4:1, 1:1, 1:2) in DMF (20 mL) for 24 h at room temperature. After dialyzing in deionized water and freeze drying, oxadiazole-decorated quaternary carboxymethyl chitosan (QCS) was obtained. ^{1}H NMR, IR spectra, UV–vis spectra was employed to determine the chemical structure of QCS.

Gelation time test

Gelation time was evaluated by the glass bottle tilting method. The solution was put in glass bottle and was observed every 15 s by tilting the glass bottle (30°). The gelation time was determined when the solution stopped flowing, suggesting the success of forming hydrogel, each group had three parallel samples.

Swelling ratio test

In the swelling rate experiment, lyophilized hydrogel specimens were used for the test, the original weight was marked as W_0, rGB/QCS/PDA-PAM hydrogel were immersed in PBS solution at 37°C and then taken out to weight (W_1) at scheduled time, swelling rate = (W_1- W_0 / W_0) × 100%.

Bacterial capture, antibacterial activity and mechanism

Bacterial adhesion capacity tests: Briefly, 1.0 mL bacteria at working concentration (OD_{600} = 1.0) were co-cultured with PDA-PAM hydrogel, QCS/PDA-PAM hydrogel, rGB/PDA-PAM hydrogel, rGB/QCS/PDA-PAM hydrogel (100 mg) at 37 ˚C for 1 h under static conditions, respectively. Then, the OD_{600} of the mixture supernatant was measured using a UV-Vis spectrophotometer, and MRSA and E. coli co-cultured with PBS ware set as the control group. The adhesion rate of bacteria (%) = (OD_{PBS} – $OD_{hydrogel}$)/OD_{PBS} × 100%. Where $OD_{hydrogel}$ was the OD_{600} of bacteria after treatment with hydrogels, and the OD_{PBS} was the OD_{600} treatment with PBS.

Antibacterial activities tests: Antibacterial activity of rGB/QCS/PDA-PAM hydrogel toward MRSA and E. coli was evaluated using colonies forming units (CFU). Specifically, PDA-PAM hydrogel, QCS/PDA-PAM hydrogel, rGB/PDA-PAM hydrogel, rGB/QCS/PDA-PAM hydrogel (size: 10 mm ×10 mm× 1 mm) were co-cultured with bacteria (1 × 10^6 CFU mL^{-1}) for 6 h at 37 ℃, respectively. During co-culturing, the four groups were exposed to NIR laser (808 nm, 0.8 W cm^{-2}) for 600s. Then, 10 μL bacterial solution of each group was uniformly spread on agar plate, which were cultured in the incubator for 14 h at 37 °C. Bacterial colony forming units were photographed to evaluate the inhibitory effect.

Bacterial morphological characterization: The morphology of hydrogels treated bacteria was observed by scanning electron microscope (SEM, Quanta 200, FEI, Holland). Briefly, bacteria were incubated with PDA-PAM hydrogel, QCS/PDA-PAM hydrogel, rGB/PDA-PAM hydrogel, rGB/QCS/PDA-PAM hydrogel for 6 h, respectively. Then, bacteria were fixed with 2.5% glutaraldehyde for 2 h, then dehydrated using gradient ethanol, lyophilized for SEM observation.

Live/dead Staining: Antibacterial efficiency of hydrogel was further checked by live/dead fluorescent staining with SYTO-9 (green fluorescence) and propidium iodide

(PI, red fluorescence). Bacteria (1×10^6 CFU mL^{-1}) were incubated with PDA-PAM hydrogel, QCS/PDA-PAM hydrogel, rGB/PDA-PAM hydrogel, rGB/QCS/PDA-PAM hydrogel for 6 h, respectively. During co-culturing, the four groups were exposed to NIR laser (808 nm, 0.8 W cm^{-2}) for 600s. After 6 h co-culture, live/dead staining was performed on bacteria and they were imaged by fluorescence microscope.

Membrane Potential of Bacteria: DiOC2(3) probe was used to measure the membrane potential of the bacteria. Bacteria (1×10^8 CFU mL^{-1}) were incubated with PDA-PAM hydrogel, QCS/PDA-PAM hydrogel, rGB/PDA-PAM hydrogel, rGB/QCS/PDA-PAM hydrogel for 6 h, respectively. During co-culturing, the four groups were exposed to NIR laser (808 nm, 0.8 W cm^{-2}) for 600s. Then, the bacteria were incubated with 30 mM DiOC2(3) for 30 min at 37 °C in darkness. The intensity of relative red and green fluorescence intensity was determined using flow cytometer.

ATP level test: ATP level in bacteria was measured using the ATP assay kit (Beyotime, China). Briefly, bacteria (1×10^8 CFU mL^{-1}) were incubated with PDA-PAM hydrogel, QCS/PDA-PAM hydrogel, rGB/PDA-PAM hydrogel, rGB/QCS/PDA-PAM hydrogel for 6 h, respectively. During co-culturing, the four groups were exposed to NIR laser (808 nm, 0.8 W cm^{-2}) for 600s. Bacteria were collected by centrifugation, and subsequently treated with the lysozyme solution (300 μL, 20 μg μL^{-1}) at 37 °C for 30 minutes to completely lyse bacteria cells. The bacterial lysate was sonicated by ultrasound for 5 min in an ice bath (30% power, 3s on, 5s off), then, was centrifuged at 8000 rpm for 5 min. Finally, bacterial supernatant (25 μL) was added into 100 μL ATP working solution, ATP chemiluminescence was detected using microplate reader.

Intracellular ROS level: ROS level in bacteria was measured using the ROS probe (DCFH-DA). Bacteria (1×10^8 CFU mL^{-1}) were incubated with PDA-PAM hydrogel, QCS/PDA-PAM hydrogel, rGB/PDA-PAM hydrogel, rGB/QCS/PDA-PAM hydrogel for 6 h, respectively. During co-culturing, the four groups were exposed to NIR laser (808 nm, 0.8 W cm^{-2}) for 600s. Bacteria were collected and incubated with serum-free medium containing DCFH-DA (Beyotime, China) for 20 min. DCF fluorescence intensity was determined by flow cytometry.

Leakage of DNA and RNA: Bacteria (1×10^8 CFU mL^{-1}) were incubated with

PDA-PAM hydrogel, QCS/PDA-PAM hydrogel, rGB/PDA-PAM hydrogel, rGB/QCS/PDA-PAM hydrogel for 6 h, respectively. During co-culturing, the four groups were exposed to NIR laser (808 nm, 0.8 W cm^{-2}) for 600 s. Bacterial suspensions were centrifuged and the supernatants were collected. A 0.22 μm membrane was used to filter any bacteria remained in the supernatant. Finally, the leakage of intracellular DNA and RNA was studied by measuring the OD_{260} value of each sample using UV–vis measurement.

In Vitro Cytotoxicity, Cell migration, Hemolysis and Hemostasis tests

Cytotoxicity: The hydrogels (size: 2 mm×2 mm×1 mm) were soaked in 75% ethanol for sterilization for 2 h for sterilization, then hydrogels were washed with PBS for 5 times to remove ethanol and transferred to a sterile 24-well plate. L929 fibroblast cell were seeded into hydrogels as a cell density of 2×10^4 /well, placed in carbon dioxide cell incubator (37°C, 5% CO_2) and continued to culture until the cells returned to the normal adherent state. A control group without hydrogel samples was conducted. The cells were stained using the Calcein-AM/PI live/dead cell double staining kit (Solarbio, CA1630) and cultured in an incubator for 30 min. The cell status was observed under an inverted fluorescent microscope (DMI8-DFC450C) to evaluate cell viability. And then the cell density was calculated by Image J software.

Cell proliferation: L929 cells were seeded in a 96-well plate with a density of 1.5×10^4 /well via cell counting kit-8 reagent (CCK-8, Dojindo, Japan),10% CCK-8 reagent was added to the corresponding wells at different time points (Day 1, 2, and 3) and the incubation was continued for 1 hours. After incubation for 1 h, the OD value of suspension at 450 nm was determined by microplate reader (SYNERGY H1, USA Bioket) and the cell viability was calculated.

Cell migration: The migration of HUVECs and L929 cells. Briefly, rGB/QCS/PDA-PAM hydrogel was spread on Petri dish and then HUVCs or L929 cells were seeded on the rGB/QCS/PDA-PAM hydrogel with a density of 1.5×10^4 /well. After culturing for 24 h, cell layer was formed on dishes. The tip of a plastic pipette was used to gently scratch the confluent cells layer to create a scarification. After cultured for another 12 h, and the cell migration was observed via optical microscope

(Leica DMi8) and the migration ratio was calculated by comparing the scarification width before and after cell migration. Cells cultured in dishes without hydrogel were used as control.

Hemolysis: The hemocompatibility of rGB/QCS/PDA-PAM hydrogel was evaluated by in vitro hemolysis assay. Fresh whole blood was acquired from rat's. After centrifugation at 1500 rpm for 600 s, the upper layer fluid was discarded and the RBCs were separated from the blood. which was washed to obtain the red blood cells (RBC) suspension. Then, the RBC was diluted by PBS buffer (ratio of 9:1). 0.2 mL RBC suspension was mixed with 5 mL PBS buffer (negative control group), 5 mL distilled water (positive control group). The rGB/QCS/PDA-PAM hydrogel (10 mg) was added to the RBCs suspension (0.2 mL RBCs suspension and 5 mL PBS buffer). All groups were incubated at 37 °C for 1 h. After culture, the mixture was centrifugated at 1500 rpm for 600 s, and the supernatant was carefully removed and transferred to 96-well plates. The optical density (OD) was determined by spectroscopy at 540 nm using microplate reader (SYNERGY H1, USA Bioket). The hemolysis ratio was calculated by hemolysis (%) = [OD value (rGB/QCS/PDA-PAM hydrogel)-OD value (negative control group)]/[OD value (positive control group)-OD value (negative control group)] × 100%.(n=3)

Hemostatic performance of hydrogels in vivo: Hemostatic performance of hydrogel in vivo was evaluated by hepatic hemorrhage model and tail hemorrhage model. Before surgical procedure, pieces of dry filter paper were weighed. After anesthesia, mice were fixed on a flat plate with an inclination angle of 30°. The liver was then pierced with a needle to bleed, after operation, the control group received no treatment, and the experimental group were immediately treated with gauze or rGB/QCS/PDA-PAM hydrogel. Hemostasis time and blood loss in each group were recorded. In the model of tail amputation, the tail was cut off from the middle with surgical scissors to cause massive bleeding. The hemostatic time and blood loss of each group also were recorded.

In Vitro Blood Coagulation Test: In vitro hemostasis performance of the hydrogel was evaluated with the coagulation time of whole blood. Primarily, rGB/QCS/PDA-

PAM hydrogel (5 × 5 × 5 mm^3), Gauze (5 × 5 × 5 mm^3) and control group was placed in clean centrifugal tube with anticoagulant whole blood (1 mL). $CaCl_2$ solution (100 μL, 0.2 M) was added and immediately activated for timing. The status was observed every 15 s by tilting the centrifuge tube (30°). The clotting time was determined as the moment when the blood had stopped flowing. Each group consisted of three parallel samples.

In Vitro Blood Clotting Index (BCI): Briefly, rGB/QCS/PDA-PAM hydrogel (5 × 5 × 5 mm^3) and Gauze (5 × 5 × 5 mm^3), blank group was put into 5 mL centrifuge tube. Then, 10 μL the whole blood with 1 μL $CaCl_2$ solution (0.2 M) was dispensed on the surface of the hydrogel at 37℃ for 10 min. 2 mL distilled water was added to the centrifuge tube at 37℃ for 20 min, red blood cells (RBC) not entrapped in the clots were hemolyzed with distilled water. Then the absorbance of supernatant was measured at 540 nm using microplate reader (Thermo Fisher Scientific). The absorbance of whole blood in distilled water was used as the control group. Blood-clotting index (BCI) was calculated by BCI (%) = OD value (sample)/OD value (distilled water) ×100%. Each group consisted of three parallel samples.

In Vitro Attachment of Platelets: The platelets adhered onto the hydrogel surface was measured with LDH assay kit. Platelet rich plasma (PRP) was prepared by centrifugation of anticoagulated whole blood at 4000 rpm for 10 min. rGB/QCS/PDA-PAM hydrogel (5 × 5 × 5 mm^3), Gauze (5 × 5 × 5 mm^3) and blank group were put into 96-well plates, then 10 μL PRP was added and shaken for 10 min at 37°C. Non-adherent platelets were washed three times with PBS, and attached platelets were lysed in 1 mL Triton X-100 (1%) at 37°C for 1 h. The microplate reader was used to measure OD at 490 nm per LDH kit instructions. 10 μL PRP without treatment was used as the control group, and the Attachment of Platelets was calculated by OD value (sample)/OD value (control) ×100%. Each group consisted of three parallel samples.

In Vivo Healing of Full-Thickness MRSA -Infected Wounds tests

The full-thickness round skin wounds with a diameter of approximate 0.8 cm were made on depilated back skin of each rat. Then, the suspension of MRSA (100 μL, 10^6

CFU mL^{-1}) was injected into the wound to establish infective model. Mice were divided into four groups, including Control, Tegaderm, rGB/QCS/PDA-PAM hydrogel and NIR+rGB/QCS/PDA-PAM hydrogel. The NIR laser was conducted (808 nm, 0.8 W cm^{-2}) for 600 s at day 0, 1, 3, 5. During the experiment period, the healing of the wound was measured and photographed every day. The rate of wound closure at various time points was calculated with the following formula: Wound Closure (%) = $A_{n\ day}$ / $A_{0\ day}$ × 100%, where the $A_{0\ day}$ and $A_{n\ day}$ respectively represented the wound area on day 0 and day n after the treatment (n = 5, 10 and 15). At 5 days' treatment, the infectious tissues of mice were separated and homogenized in normal saline. 10 μL of the diluted solution was poured onto LB agar plate and subjected to culture for 14 h at 37°C. The grown colonies on the plate were then counted for analysis. The viability of the bacteria was calculated with the following formula: Bacterial Viability (%) =CFU_S / $CFU_{Control}$ × 100%, where the CFU_S and $CFU_{Control}$ refer to the colony forming units in the sample and control, respectively. Moreover, after 15-days treatment, Mice were sacrificed and tissues from healing wound were obtained for pathological analysis. H&E staining, Masson's trichrome staining and CD31 staining were implemented to assess the wound healing mechanism.

Figures and tables

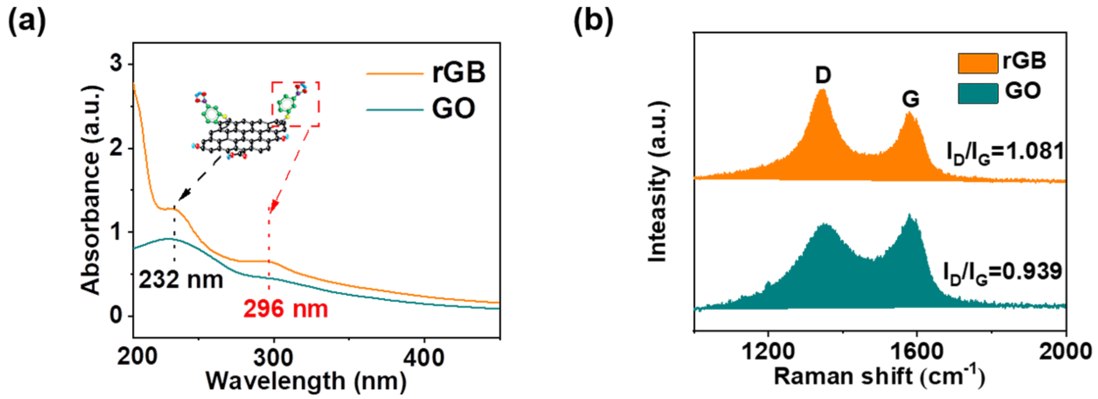

Figure S1. (a) UV–vis absorption spectra, (b) Raman spectra of GO and rGB.

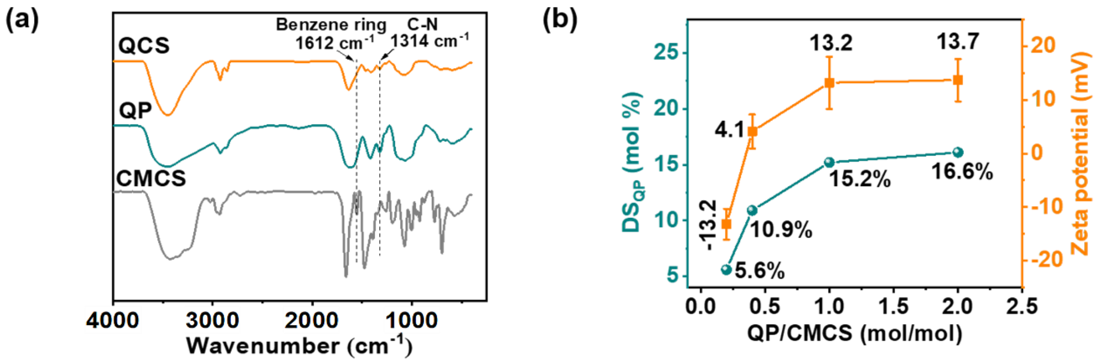

Figure S2. (a) FT-IR spectra of CMCS, QP and QCS polymer. (b) Degree and Zeta potential of QCS conjugates.

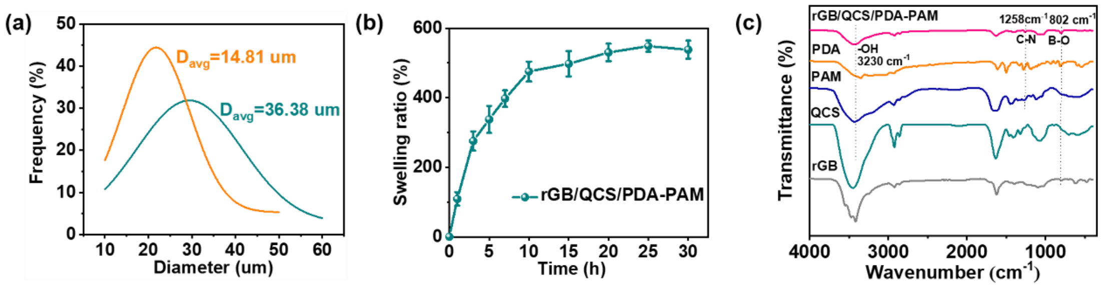

Figure S3. (a) Diameter of distributed big pores and small pores in hydrogel. (b) Swelling kinetics curves of hydrogel in PBS (pH=7.4) at 37 °C Data are presented as mean ± SDs (n = 3). (c) FT-IR spectra of rGB/QCS/PDA-PAM hydrogel.

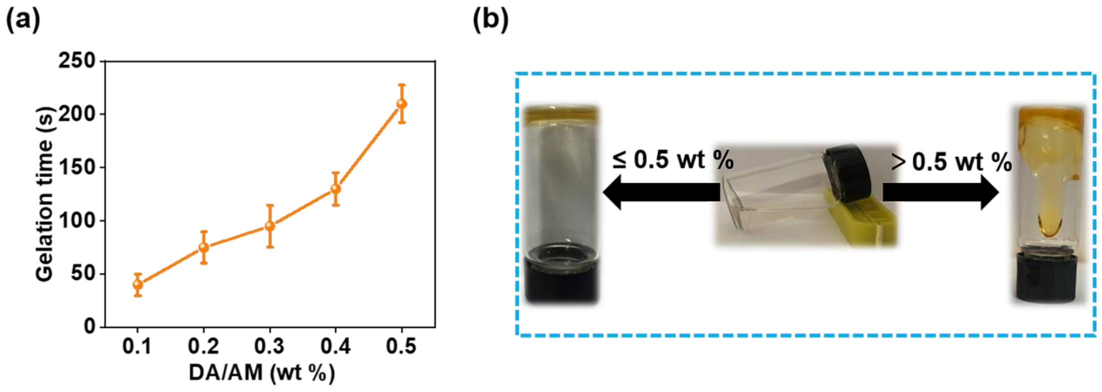

Figure S4. (a) Gelation time changes as variant DA/AM ratio for PDA-PAM hydrogel. Data are presented as mean ± SDs (n = 3). (b) The photos of PDA-PAM hydrogel formed with appropriate DA/AM interval.

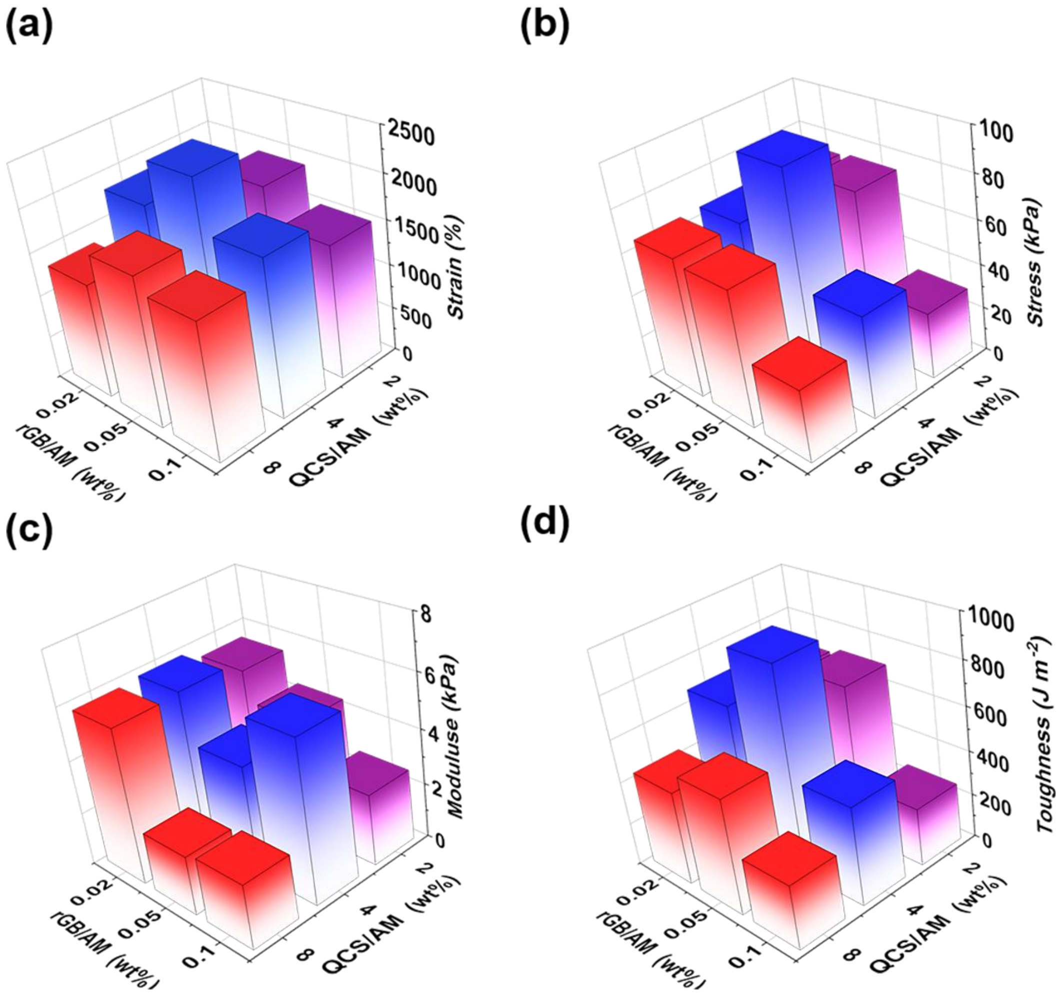

Figure S5. The (a) Strain, (b) Stress, (c) Modulus and (d) Toughness of various rGB/QCS/PDA-PAM hydrogel.

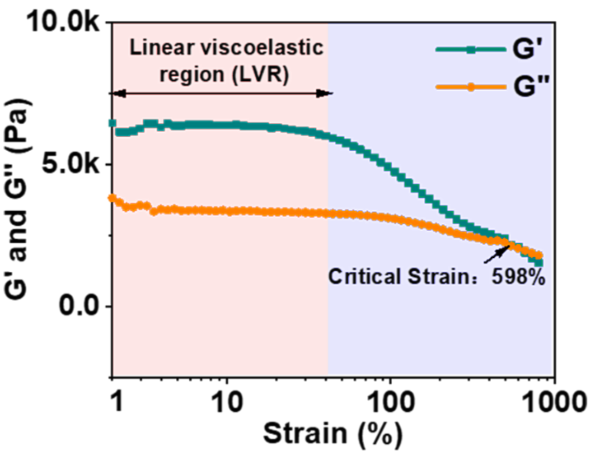

Figure S6. Strain weep curve for linear viscoelastic region and critical strain.

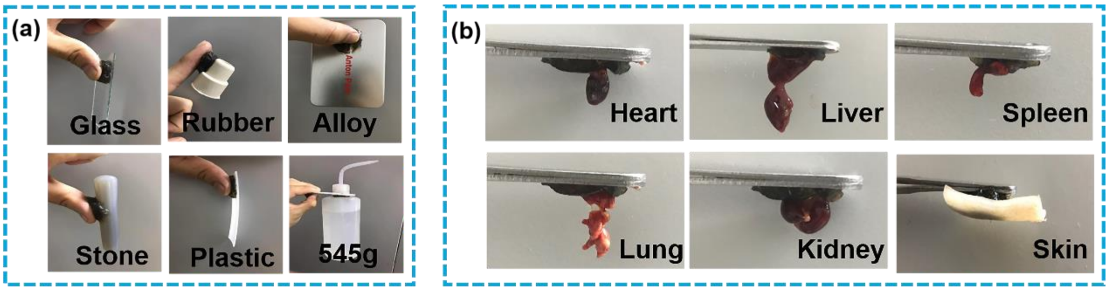

Figure S7. (a) Photographs of rGB/QCS/PDA-PAM hydrogel adhesion onto dry substrates and (b) wet tissue.

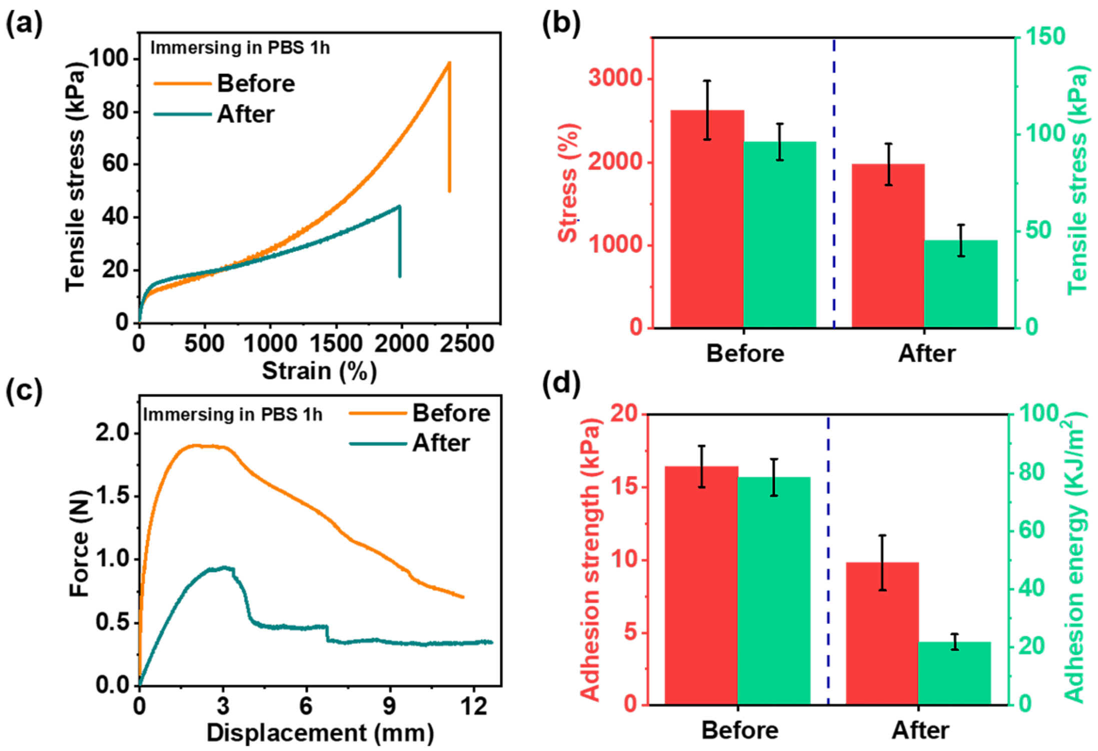

Figure S8. (a) Stress-strain curves of rGB/QCS/PDA-PAM hydrogel before and after immersing in PBS, and the corresponding (b) Strain and Tensile stress. (c) Force−displacement curves of rGB/QCS/PDA-PAM hydrogel before and after immersing in PBS on porcine skin, and the corresponding (d) Adhesive strength and Adhesion energy. Data are presented as mean ± SDs (n = 3).

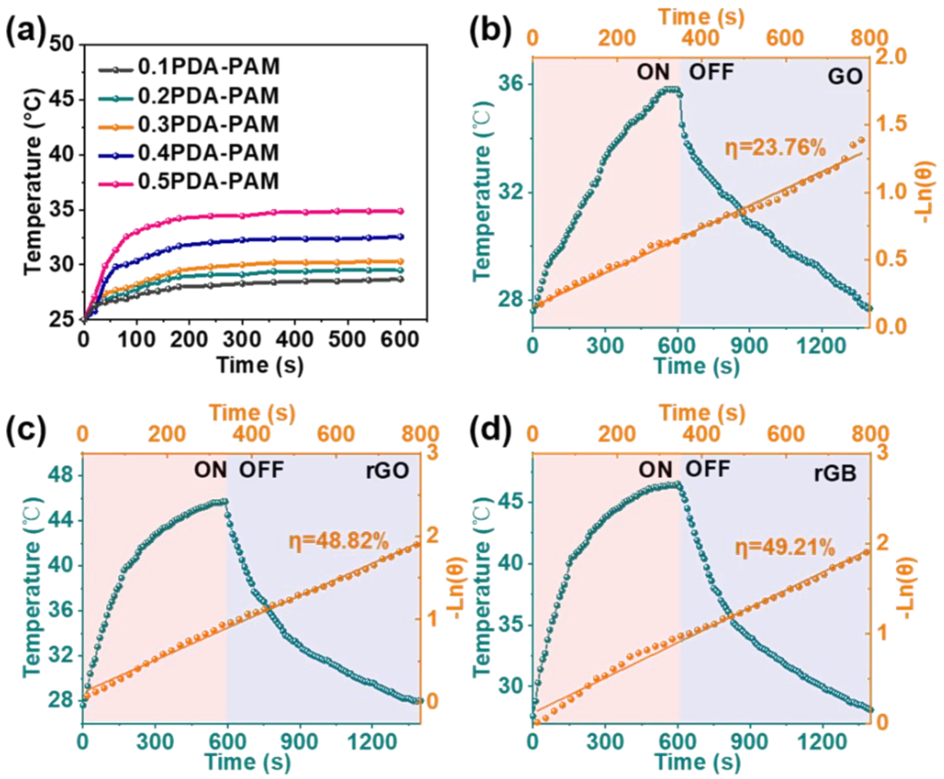

Figure S9. (a) Photothermal heating curves of PDA-PAM hydrogel with variant DA/AM ratio. Photothermal-conversion efficiency of (b) GO, (c) rGO and (d) rGB.

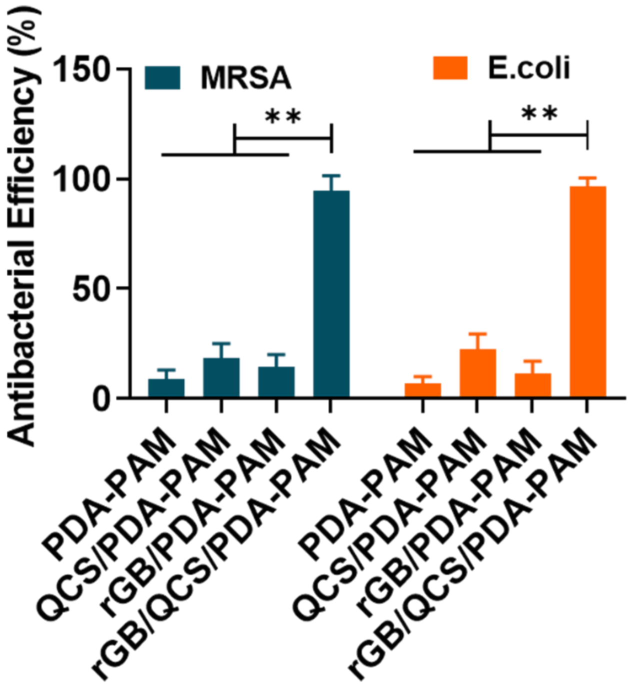

Figure S10. Quantification of the relative antibacterial efficiency after different treatments. Data are presented as mean ± SDs (n = 3), **p<0.01. Student's t-test.

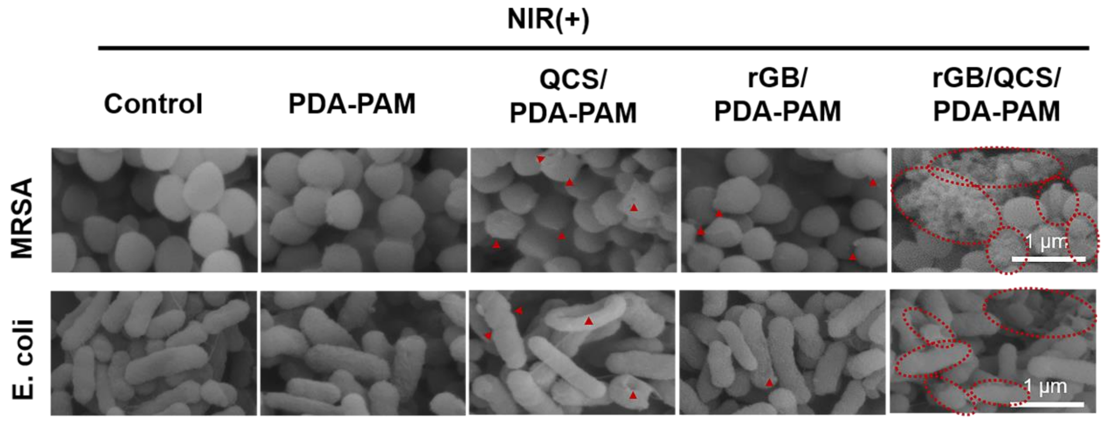

Figure S11. SEM image of MRSA and E. coli. with different treatments under mild NIR irradiation (808 nm, 0.8 W cm $^{-2}$, 600 s).

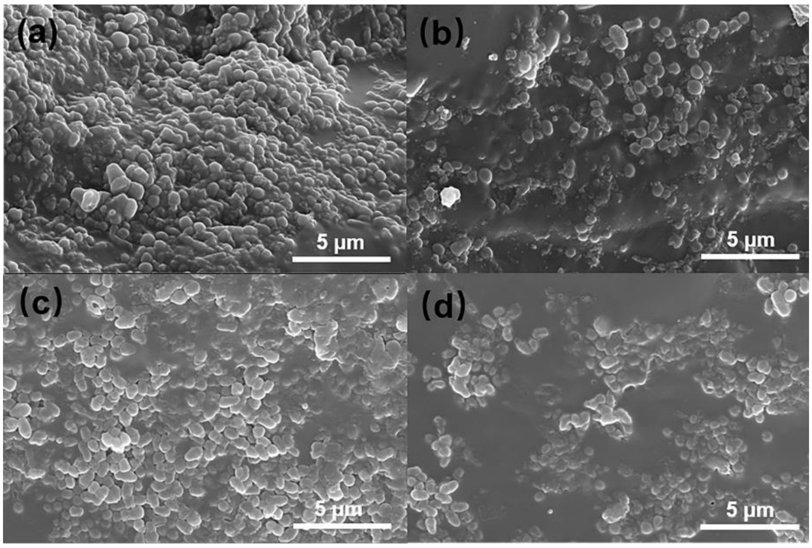

Figure S12. SEM images of MRSA co-cultured with hydrogels for (a) 1 h and (b) 6 h. SEM images of E. coli co-cultured with hydrogels for (c) 1 h and (d) 6 h.

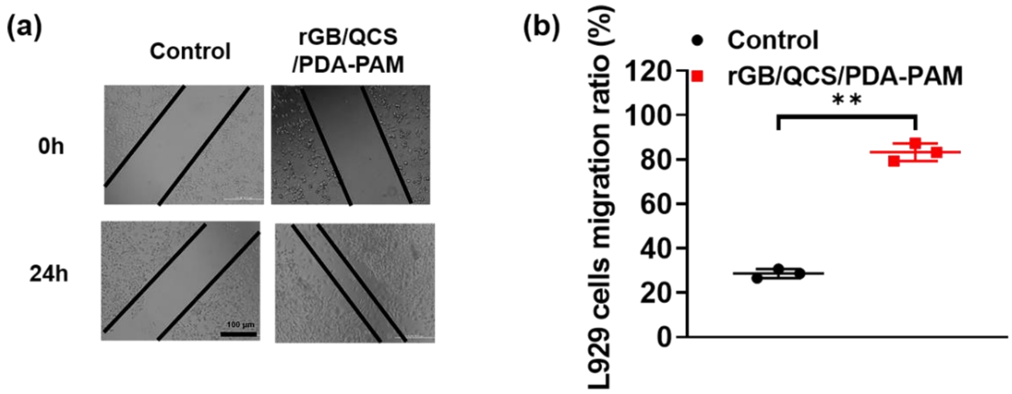

Figure S13. (a,b) Optical images and the migration ratio of L929 cells after rGB/QCS/PDA-PAM hydrogel and PBS treatment for 24 h. Data are presented as mean ± SDs (n = 3), *p < 0.05, **p<0.01. Student's t-test.

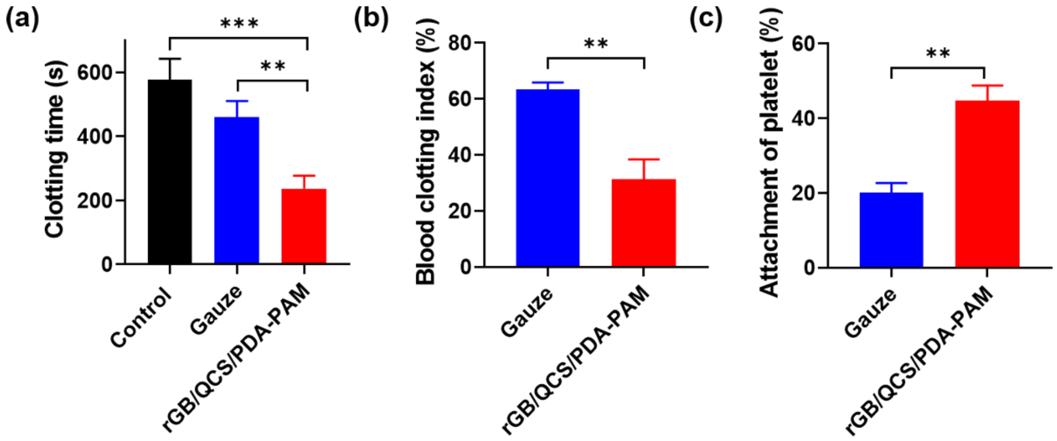

Figure S14. (a) The coagulation time of whole blood and (b) BCI of rGB/QCS/PDA-PAM hydrogel in whole blood coagulation. (c) The attachment of platelets in different groups. Data are presented as mean ± SDs (n = 3), *p < 0.05, **p<0.01 ***p < 0.001. Student's t-test.

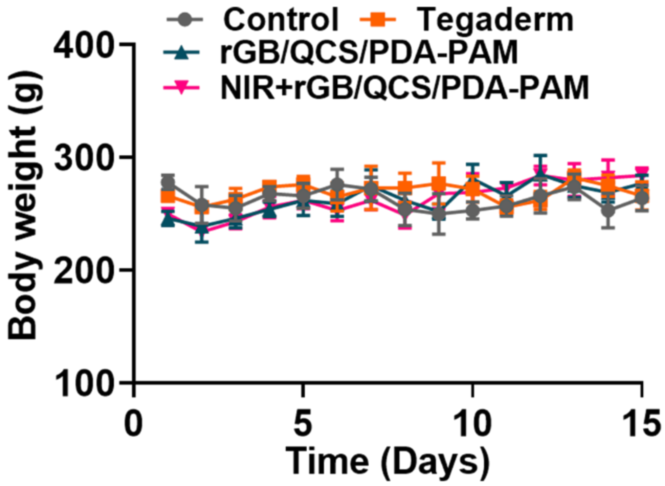

Figure S15. Changes of body weights of MRSA-infected mice with different treatments. Data are presented as mean ± SDs (n = 3).

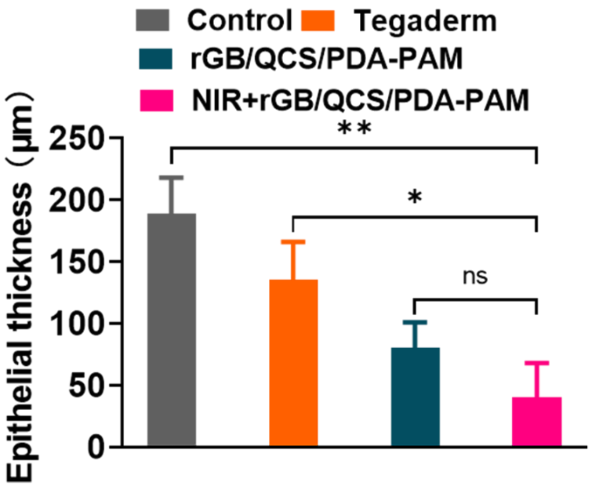

Figure S16. Epithelial thickness with different treatments after 15 days by H&E staining. Data are presented as mean ± SDs (n = 3), *p < 0.05, **p<0.01 Student's t-test.

Table S1. The DA/AM ratio in basic PDA-PAM hydrogel composition.

Hydrogels	DA/AM (wt %)	AM (g)	APS/AM (wt %)	BIS/AM (wt ‰)	TMEDA (μL)	Water (mL)
0.1PDA-PAM	0.1	1	10	0.6	5	2.5
0.2PDA-PAM	0.2	1	10	0.6	5	2.5
0.3PDA-PAM	0.3	1	10	0.6	5	2.5
0.4PDA-PAM	0.4	1	10	0.6	5	2.5
0.5PDA-PAM	0.5	1	10	0.6	5	2.5

Table S2. The rGB/AM and QCS/AM ratio in rGB/QCS/PDA-PAM hydrogel composition.

Hydrogels	rGB/AM (wt %)	QCS/AM (wt %)
0.02rGB/2QCS/0.3PDA-PAM	0.02	2
0.05rGB/2QCS/0.3PDA-PAM	0.05	
0.1rGB/2QCS/0.3PDA-PAM	0.1	
0.02rGB/4QCS/0.3PDA-PAM	0.02	4
0.05rGB/4QCS/0.3PDA-PAM	0.05	
0.1rGB/4QCS/0.3PDA-PAM	0.1	
0.02rGB/8QCS/0.3PDA-PAM	0.02	8
0.05rGB/8QCS/0.3PDA-PAM	0.05	
0.1rGB/8QCS/0.3PDA-PAM	0.1	
